# Spatiotemporal characterization of Alzheimer disease pathology in living human brain tissue

**DOI:** 10.1038/s41467-026-76494-4

**Published:** 2026-09-24

**Authors:** Aram Aslanyan, Soumya Mukherjee, Eleanor M. Moncur, Alicja Szadziewska, Yingxin He, Przemysław R. Kac, Reid Coyle, Elena Camporesi, Chihiro Sato, Nupur Ghoshal, Nicolas R. Barthélemy, Jack Wood, Junyue Ge, Maciej Dulewicz, Srinivas Koutarapu, Katherine Schwetye, Kaleigh F. Roberts, Eimear C. Murphy, Francesco Carletti, Kaj Blennow, Fernando Gonzalez Ortiz, Claire A. Leckey, Tatiana A. Giovannucci, Kanza Tariq, Henrik Zetterberg, Donald L. Elbert, Lewis Thorne, Ahmed K. Toma, Laurence Watkins, Nick C. Fox, Randall J. Bateman, Jörg Hanrieder, Ross W. Paterson

**Affiliations:** 1 Dementia Research Centre, UCL Queen Square Institute of Neurology, University College London, London, UK; 2 National Hospital for Neurology and Neurosurgery, London, UK; 3 Department of Neurology, Washington University School of Medicine in St Louis, St Louis, MO USA; 4 The Tracy Family Stable Isotope Labeling Quantitation Center, Washington University School of Medicine, Washington University in St. Louis, St. Louis, MO USA; 5 Department of Brain Repair & Rehabilitation, UCL Queen Square Institute of Neurology, University College London, London, UK; 6 Department of Psychiatry and Neurochemistry, Institute of Neuroscience and Physiology, the Sahlgrenska Academy at the University of Gothenburg, Mölndal, Sweden; 7 Department of Neuroscience, Physiology & Pharmacology, University College London, London, UK; 8 Department of Pathology and Immunology, Washington University School of Medicine in St. Louis, St. Louis, MO USA; 9 Clinical Neurochemistry Lab, Sahlgrenska University Hospital, Mölndal, Sweden; 10 Paris Brain Institute, ICM, Pitié-Salpêtrière Hospital, Sorbonne University, Paris, France; 11 Clinical Neurodegenerative Disorder Research Center, Division of Life Sciences and Medicine, and Department of Neurology, Institute on Aging and Brain Disorders, University of Science and Technology of China and First Affiliated Hospital of USTC, Hefei, P. R. China; 12 UK Dementia Research Institute at UCL, London, UK; 13 Department of Neurodegenerative Disease, UCL Institute of Neurology, Queen Square, London, UK; 14 Hong Kong Center for Neurodegenerative Diseases, Clear Water Bay, Hong Kong China; 15 Wisconsin Alzheimer’s Disease Research Center, University of Wisconsin School of Medicine and Public Health, University of Wisconsin-Madison, Madison, WI USA; 16 Department of Neurology, University of Washington, Seattle, WA USA; 17 Hope Center for Neurological Disorders, St. Louis, MO USA; 18 Charles F. and Joanne Knight Alzheimer Disease Research Center, Washington University School of Medicine, St. Louis, MO USA; 19 Darent Valley Hospital, Dartford, UK

**Keywords:** Alzheimer's disease, Diagnostic markers, Alzheimer's disease

## Abstract

Alzheimer disease begins in the brain many years before symptoms, but early changes are usually studied after death or indirectly through fluid and imaging biomarkers. Here we show that small brain biopsies collected during ventriculoperitoneal shunt surgery can be used to study Alzheimer disease pathology and protein turnover during life. We analysed biopsies from 18 individuals with suspected normal pressure hydrocephalus, alongside matched ventricular and lumbar cerebrospinal fluid and post-mortem control brain tissue. Amyloid plaques were present in 9 of 18 biopsies, while mature tau tangles were rare. Matrix-assisted laser desorption/ionization mass spectrometry imaging mapped amyloid plaque chemistry within tissue. Phosphorylated tau 217 was enriched around plaques, supporting a local relationship between amyloid and tau pathology. Stable isotope labelling, which tracks newly made proteins, detected rapid tau labelling within approximately 3 hours and estimated brain tau half-life at about 34 days. Amyloid plaques showed little detectable turnover.

## Abstract (web summary)

This study uses brain biopsies collected during shunt surgery to examine early Alzheimer disease in living brain tissue, showing rapid tau turnover and stable amyloid plaques.

## Introduction

Alzheimer disease (AD), the commonest cause of dementia, is characterised by deposition of β-amyloid (Aβ) peptide derived from amyloid precursor protein (APP) and intracellular deposition of hyperphosphorylated tau protein^1–3^. These neuropathological observations are based on *post-mortem* examination of the major pathological hallmarks, i.e. extracellular Aβ plaques; extracellular neuritic plaques; intracellular tau tangles^4,5^ and supported by cerebrospinal fluid (CSF), plasma^6–8^ and imaging biomarkers (Aβ and tau Positron Emission Tomography (PET))^9–15^.

We lack understanding of the evolution of Aβ and tau pathologies in human brain tissue, particularly at earlier stages of the AD continuum. This is due to limited access to human brain tissue during life, and limitations in plasma and CSF biomarkers for spatial and temporal biochemical characterization of earliest neuropathologic AD changes in the brain^16,17^. This is especially important as recent advancements in fluid biomarkers implicate different Aβ and phosphorylated tau (pTau) species at earlier stages of the AD continuum^18–21^.

Until recently, technical limitations have prevented detailed biochemical characterization of Aβ and tau pathological structures at cellular and sub-cellular level, with limited means of determining the rate at which proteins are synthesized, accumulate, are cleared or modified over time^22–24^. Understanding how these structures form, how they evolve over time and how Aβ and tau interact ex vivo has implications for therapeutic intervention, particularly at the earliest pre-symptomatic phase of the disease^25–28^.

A number of complementary techniques can now help to overcome these problems. Immunoprecipitation (IP) combined with targeted mass spectrometry (MS) can provide high resolution and detailed information on the biochemical changes in the protein when soluble and insoluble protein fractions are obtained. However, it has its own challenges such as poor spatial visualisation^29^. Stable Isotope labelling kinetics (SILK) uses isotopically labelled amino acids that are intravenously administered to humans to track protein kinetics^30–32^. They pass freely across the blood-brain barrier and become incorporated into proteins translated in the central nervous system. Making a ratio between labelled and unlabelled proteins in brain tissue, ventricular and lumbar CSF and other physiological compartments permits them to be ‘dated’ for quantitation of production and clearance rates. This technique enhances temporal resolution; however it compromises on fewer peptides being available for measurement^30–32^. Enhanced spatial resolution has also recently been achieved by matrix-assisted laser desorption/ionization (MALDI) mass spectrometry-based imaging (MSI) which has the chemical specificity and spatial resolution for the assessment of local peptide metabolism in situ at the low micrometre scale^33^. We and others have leveraged these technical advances to describe the chemical changes in the brains of AD mice labelled with a ^15^N stable isotope and in human *post-mortem* brain tissue^34,35^.

Further information about protein inclusions can be gleaned from quantitation of post-translational modifications (PTMs). Tau becomes phosphorylated and specific phosphorylation sites can be a valuable predictor of disease stage in blood and CSF^36^. Aβ, a long-lived peptide aggregating in the brain, undergoes multiple PTMs (e.g. sequential N-terminal truncations, pyroglutamate formation, nitration, phosphorylation, dimerization, citrullination and Asp residue isomerisation among others)^19,37–43^. Isomerised Aβ in the brain can distinguish the status of amyloidosis in the AD spectrum and is useful for predicting plaque maturity^18,44^.

Therefore, exploring the protein structures in more depth is both important and technically feasible, but it is still difficult to study human brain tissue during life due to obvious technical and ethical restrictions. To understand the structure and evolution of plaques and tangles ex vivo we sought to identify individuals who could donate brain tissue during life. We enroled a population of individuals undergoing neurosurgery for other clinical indications in a population enriched with AD pathology and labelled them with stable isotope labelled amino acid prior to their procedure. We aimed to determine *what* tau and Aβ species are found ex vivo, *when* they are synthesized and incorporated into pathological inclusions, *where* pathological localization and PTMs occur and how pathologies interact, using SILK combined with other tissue imaging and biochemical characterization techniques.

## Results

### Early brain amyloid and tau deposition can be chemically characterised in parieto-occipital cortex

We collected ex vivo brain biopsies from individuals with suspected normal pressure hydrocephalus (NPH) (*n* = 18) undergoing ventriculoperitoneal shunt (VPS) placement, providing a rare opportunity to evaluate tissue pathology ex vivo. Post-mortem control brain samples were available from individuals with pathological diagnoses of AD (*n* = 2), Corticobasal Degeneration (CBD) (*n* = 1), healthy controls (*n* = 2) and amyloid positive participants without cognitive symptoms at death (*n* = 2)^45^ (Supplementary Table 1). NPH subjects were in between 7th and 9th decade of life (median was 8th decade of life) and were predominantly male (2:1 ratio) (Table 1 and Supplementary Table 2). They mainly presented with gait and balance problems and/or falls (94%) and urinary symptoms (77.8%). On assessment they all had cognitive symptoms with episodic and executive domains being most affected, and their Mini-Mental State Examination (MMSE) ranged between 21-30/30. In response to CSF diversion therapy, 13/18 had initial improvement in gait. Detailed clinical information can be found in Supplementary Table 2.

**Table 1:** Participant characteristics, CSF biomarker profiles and neuropathology results of individuals undergoing brain biopsy

	Clinical Details			Cerebrospinal Fluid Biomarkers					Neuropathology	
	Decade of life	MMSE	Time from labelling to brain biopsy	Aβ1-42/40 ratio (>0.072)	pTau217 *	pTau181 *	T-Tau *	Neurofilament light *	Amyloid plaque density (plaque count / mm²)	Tissue area positive for pTau217 staining (%)
**Subject 1**	7th	29/30	8 h 18 min	0.086	14.6	834.9	795.4	522.7	0	0.00
**Subject 2**	9th	29/30	5 h	0.078	9.4	756.7	550.1	528.2	1	0.00
**Subject 3**	8th	26/30	11 h 42 min	0.065	10.2	862.9	1021.0	353.9	37	1.28
**Subject 4**	8th	24/30	5 h 33 min	0.085	26.2	1198.5	2306.5	768.5	3	0.14
**Subject 5**	7th	26/30	128 days	0.084	3.6	141.1	59.8	1717	0	0.01
**Subject 6**	8th	28/30	42 days	0.093	7.3	219.9	100.0	1558	0	0.12
**Subject 7**	8th	30/30	Unlabelled	0.058	7.3	595.3	620.7	458.9	2	0.01
**Subject 8**	8th	30/30	Unlabelled	0.060	3.5	510.6	286.1	293.3	1	0.02
**Subject 9**	8th	26/30	Unlabelled	0.128	7.7	773.5	1099.0	525.5	0	0.02
**Subject 10**	9th	24/30	Unlabelled	0.039	24.6	1614.2	1269.9	1468.8	4	0.02
**Subject 11**	8th	24/30	Unlabelled	0.039	22.6	884.1	621.5	918.3	43	0.06
**Subject 12**	9th	24/30	Unlabelled	0.042	20.7	1183.8	1713.9	637.8	37	1.03
**Subject 13**	9th	21/30	Unlabelled	0.083	6.3	555.3	312.4	686.6	0	0.11
**Subject 14**	8th	29/30	4 h 16 min	0.077	118.8	2942.5	3295.3	475.5	0	0.13
**Subject 15**	8th	25/30	3 h 31 min	0.072	62.1	1339.8	1497.3	407.2	0	0.68
**Subject 16**	8th	21/30	3 h 17 min	0.058	30.5	725.1	768.9	491.7	15	0.15
**Subject 17**	8th	28/30	4 h 54 min	0.070	14.1	786.9	611.9	440.0	0	0.01
**Subject 18**	8th	30/30	4 h 24 min	0.085	30.9	996.5	1103.2	509.3	0	0.07

All had suspected NPH. *pTau217, pTau181, T-Tau and Neurofilament light assays (AlzPath and Quanterix) do not have accepted normal range in CSF.

*Aβ* β-amyloid, *CSF* cerebrospinal fluid, *MMSE* Mini Mental State Examination, *NPH* Normal Pressure Hydrocephalus, *pTau181* phosphorylated Tau181, *T-Tau* Total Tau.

**Fig. 1:**
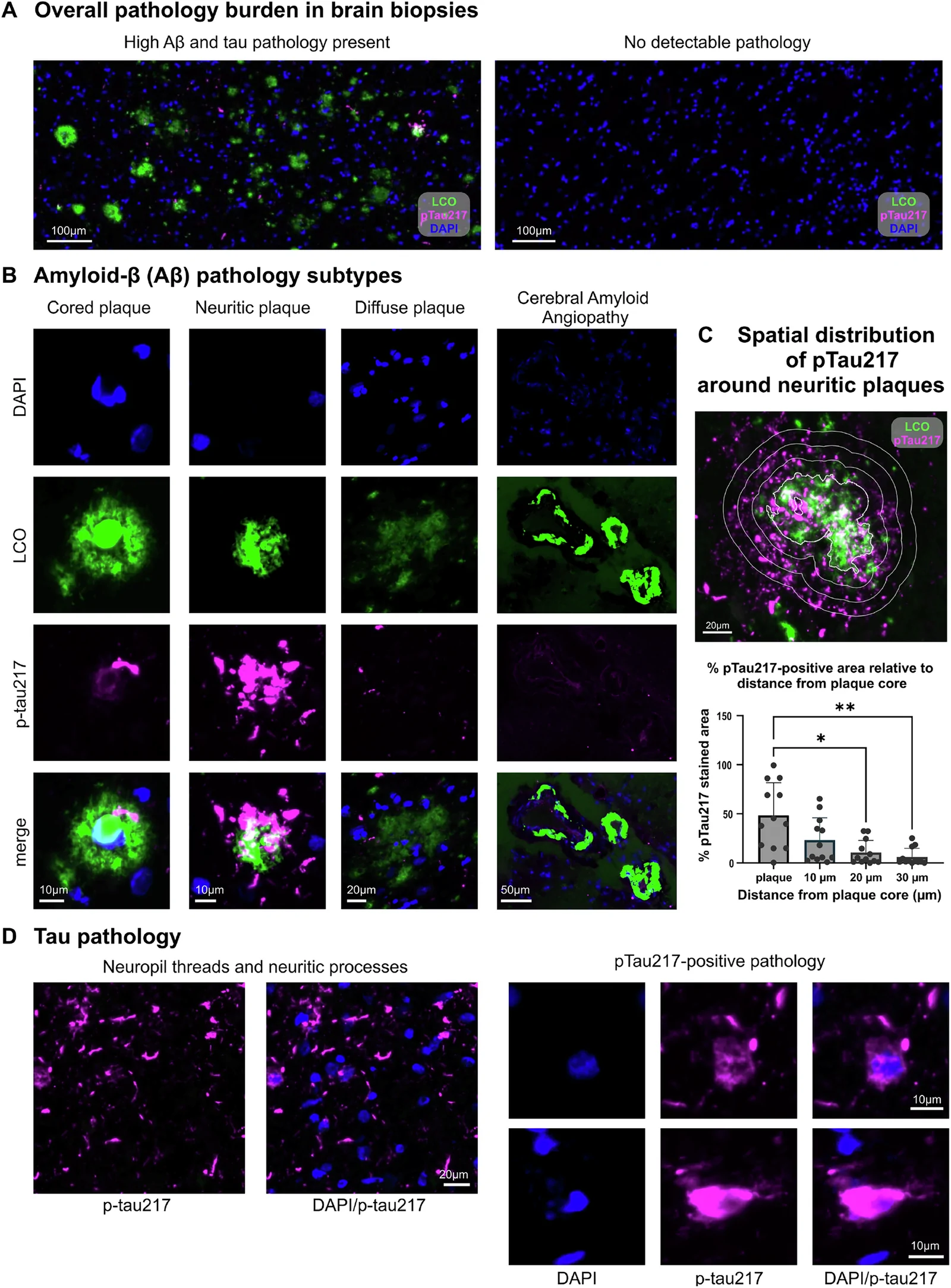
Characterization of AD-type pathology in brain biopsy samples. **A** Representative immunofluorescence images showing the range of AD-type pathology observed across biopsy samples. Amyloid deposits were visualized using LCO staining (green), phosphorylated tau (pTau217) was detected by immunofluorescence (magenta), and nuclei were counterstained with DAPI (blue). **B** Examples of distinct Aβ pathology subtypes identified by LCO staining: cored plaques, neuritic plaques, diffuse plaques, and cerebral amyloid angiopathy. Neuritic plaques exhibited prominent co-localization of LCO and pTau217, indicating the presence of phosphorylated tau-positive dystrophic neurites surrounding amyloid deposits. **C** Quantitative analysis of pTau217 distribution around neuritic plaques revealed a significant enrichment of tau immunoreactivity within the plaque, which progressively decreased with increasing distance from the centre. A total of 12 neuritic plaques from 3 independent pTau217-positive subjects were analyzed, with one biopsy sample available per subject. Individual plaques were used as the unit of analysis. Data are presented as mean ± SD. Statistical significance was assessed across distance groups using a Kruskal-Wallis test followed by Dunn’s multiple-comparisons test. Adjusted p values were as follows: plaque vs. 10 µm, *p* = 0.7765; plaque vs. 20 µm, *p* = 0.0172; plaque vs. 30 µm, *p* = 0.0012; 10 µm vs. 20 µm, *p* = 0.8569; 10 µm vs. 30 µm, *p* = 0.1630; and 20 µm vs. 30 µm, *p* > 0.9999. **D** Tau pathology was primarily limited to neuropil threads and neuritic processes, with occasional pTau217-positive neuronal inclusions exhibiting early neurofibrillary tangle-like morphology in selected cases. Images shown in panels A, B, and D are representative of observations from 18 independent subjects, with one biopsy sample available per subject and 1–3 tissue sections analyzed per biopsy. Multiple sections from the same biopsy were considered within-subject observations and were not treated as independent biological replicates. Source data are provided as a Source Data file. AD Alzheimer disease, Aβ, β-amyloid, DAPI 4′,6-diamidino-2-phenylindole, LCO luminescent conjugated oligothiophene, pTau217 phosphorylated tau217.

AD pathology was evaluated in brain biopsy samples using immunofluorescence (IF) and luminescent conjugated oligothiophene (LCO) staining^46^. Aβ pathology was detected in half of biopsy samples, with variable plaque burden across subjects. Cored plaques predominated, although diffuse and neuritic forms (associated with pTau217-positive neurites) were also observed. Quantitative analysis revealed the highest amyloid density in subjects 3, 11, 12, and 16. Subject 16 demonstrated moderate amyloid deposition together with evident cerebral amyloid angiopathy (CAA), characterised by amyloid deposition in vessel walls (Table 1; Fig. 1).

Tau pathology was overall limited in extent (Table 1). Across the cohort, phosphorylated tau (pTau217) immunostaining revealed mainly neuritic threads and tau associated with plaques (neuritic plaques), rather than mature neurofibrillary tangles. In subjects 3 and 12, occasional compact tau-positive aggregates were observed that may represent early tangle formation. Tau burden in the tissue was quantified from immunofluorescence images as the proportion of pTau217-positive area relative to the total tissue area analysed, providing a percentage estimate of tau deposition. Overall, tau pathology was low across most cases, with values ranging from 0.00 to 1.28%. The highest tau burden was observed in subject 3 (1.28%) and subject 12 (1.03%), both of which also exhibited dense amyloid deposition.

Further spatial analysis of pTau217 distribution around neuritic plaques revealed a pronounced enrichment of tau immunoreactivity within the plaque area, with signal intensity progressively decreasing with increasing distance from the plaque (Fig. 1C). This pattern indicates that tau accumulation was closely associated with amyloid plaques.

Nine of the 18 individuals with suspected NPH had ventricular CSF biomarker profile suggesting brain amyloidosis measured by SIMOA (Table 1). Of these, two participants had positive CSF results with no evidence of plaques in their respective brain samples (Subjects 15 and 17) and additional two CSF results were negative with evidence of plaques in their respective brain samples (Subjects 2 and 4). Other ventricular CSF biomarkers (pTau217, pTau181, total Tau and NfL) did not show correlation with plaque load.

**Fig. 2:**
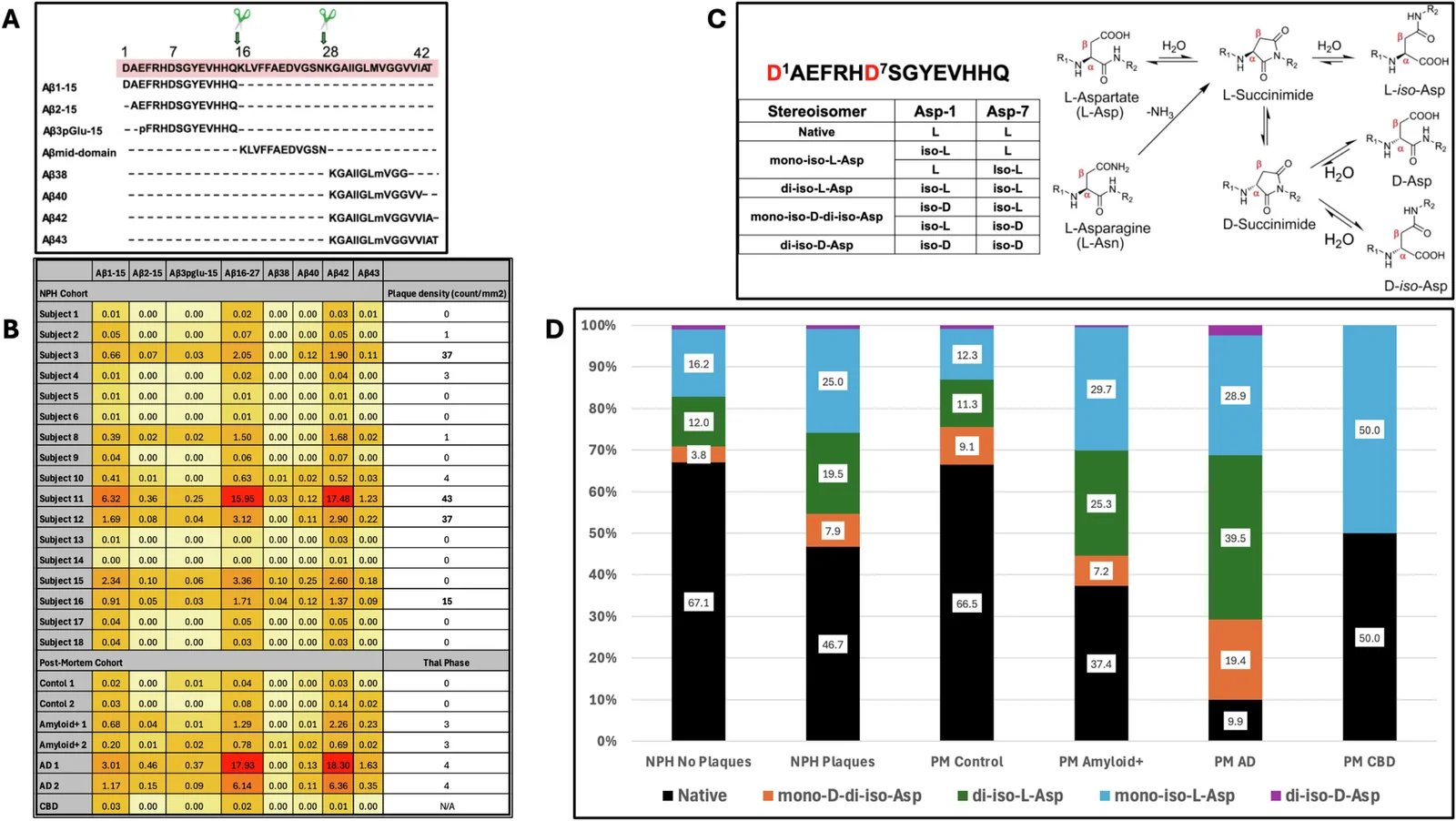
Isomerised Aβ species quantified by IP-MS in NPH and *post-mortem* brain. **A** Common Aβ isoforms identified using IP-MS. **B** Heatmap of absolute abundance of Aβ isoforms (ng/mg brain) with corresponding plaque density (counts/mm^2^). The highest Aβ burden is seen in Subject 11 (highest plaque density) and AD1 *post-mortem* control. In general, the most abundant isoforms were Aβ42 and Aβ16-27. **C** Schematic of Aβ Asp1 and Asp7 isomerisation pathways and representative structures for Aβ1-15 isomerised species. **D** Average proportions of Aβ_1-15_ Asp1,7 isomerisation states in NPH brain biopsies and post-mortem (PM) controls. Highest proportions of non-native isomers are observed in PM AD, PM Amyloid+ and NPH Plaques. Source data are provided as a Source Data file. AD Alzheimer disease, Aβ β-amyloid, Amyloid +  amyloid positive, CBD corticobasal degeneration.

The relative abundance of Aβ isoforms in the insoluble fraction of brain tissue is shown in Fig. 2B. The most abundant isoforms were Aβ42 and Aβ16-27. As expected, all Aβ isoforms were more abundant in the NPH case with the highest plaque burden and the post-mortem AD and Aβ-positive controls compared to post-mortem controls and NPH cases with lower plaque burden.

**Fig. 3:**
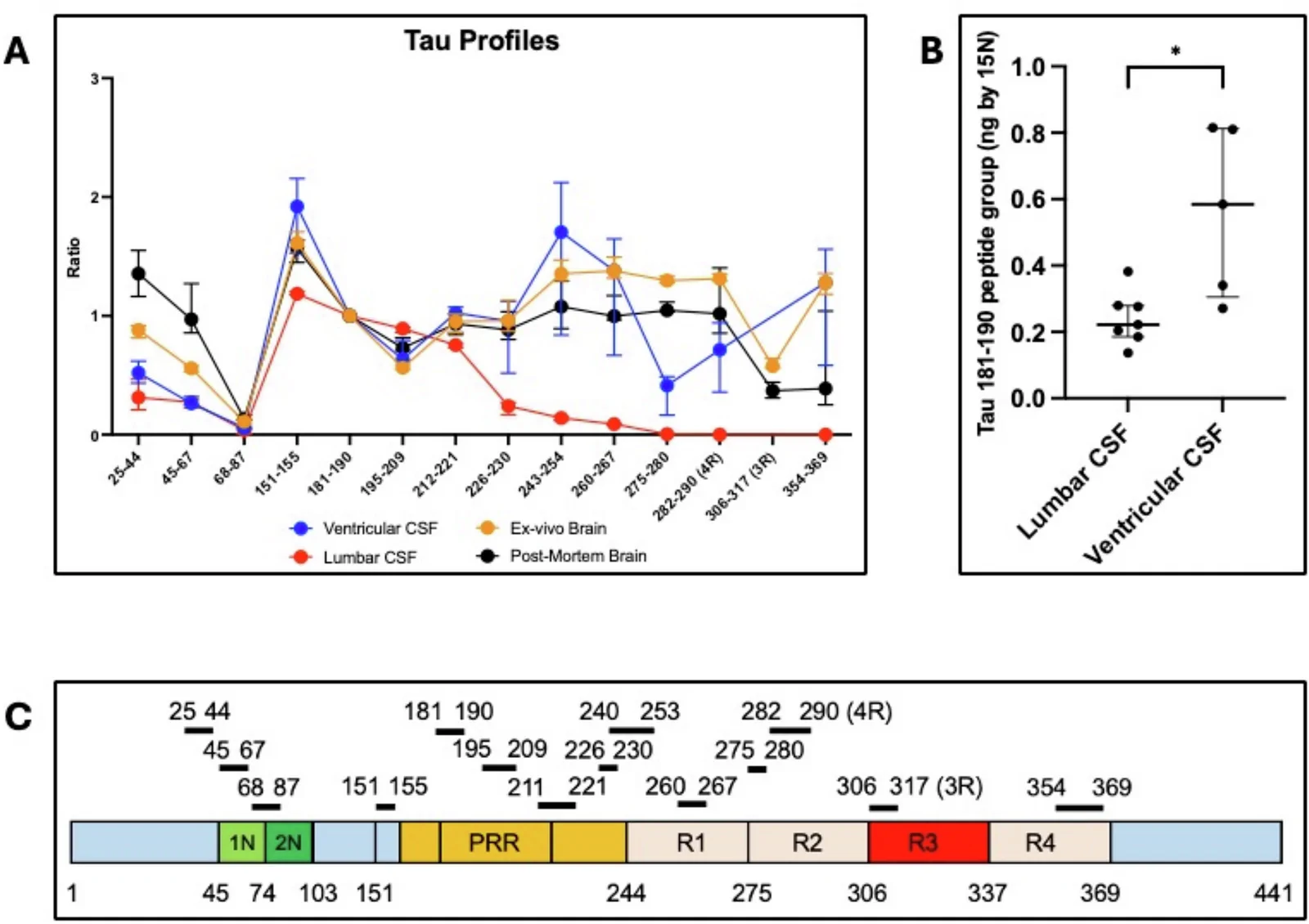
Tau peptide group profiles across CNS compartments. **A** Tau peptide group profile measured by IP-MS in ex vivo brain (ng/mg brain) (*n* = 6), *post-mortem* brain (ng/mg brain) (*n* = 7), ventricular (*n* = 5) and lumbar (*n* = 7) CSF (ng by 15 N). Here, n denotes independent biological subjects. Values are shown in ratios normalised to their respective peptide group 181–190. Points represent the median across biological samples for each compartment and error bars indicate the IQR. Compared with *post-mortem* brain, ex vivo brain showed lower relative abundance at N-terminal peptide groups 25-44 and 45-67 (two-sided Mann–Whitney U; Benjamini-Hochberg correction across *n* = 14 peptide groups; adjusted *p* = 0.008 and 0.006, respectively). In contrast, several C-terminal peptide groups (260–267, 275–280, 354–369) were relatively higher (adjusted *p* = 0.006, 0.006, and 0.013). Lumbar CSF displayed a distinct pattern, peaking at 151-155 with low relative abundance toward the C-terminus, whereas ventricular CSF resembled the brain profiles with peaks at 151–155, 243–254, 354–369. **B** Absolute tau 181-190 (TPPS, ng by 15 N) in NPH ventricular (*n* = 5) and lumbar (*n* = 7) CSF. Dots represent individual biological subjects; horizontal bars show median and IQR. Ventricular CSF values were higher than lumbar CSF (two-sided Mann–Whitney U, *p* = 0.030). **C** Schematic of human tau protein sequence showing N-terminal inserts (1 N/2 N), the proline-rich region (PRR), and microtubule-binding repeat domains (R1-R4). Black bars indicate the tau sequence regions. Source data are provided as a Source Data file. CNS Central Nervous System, IP-MS Immunoprecipitation-Mass Spectrometry, IQR Interquartile Range, NPH Normal Pressure Hydrocephalus.

Further tau analysis focused on the soluble fraction of brain homogenate. We characterised tau using high resolution IP-MS and compared the relative abundance of peptides across the full length of tau in ex vivo brain, ventricular and lumbar CSF (Fig. 3A). We were also able to compare ex vivo brain tau profiles with those from *post-mortem* brain tissue. Overall, the profiles were similar, but we found a higher abundance of peptide groups 25–44 and 45–67 (adjusted *p* = 0.008 and 0.006, respectively) and lower abundance of fragments containing amino acids 260-267, 275-280 and 354–369 (adjusted *p* = 0.006, 0.006, 0.013) in the post-mortem samples (Fig. 3). We then compared ex vivo brain tau profile with ventricular and lumbar CSF profile (Fig. 3) and found that the ventricular CSF profile was similar to the brain profile peaking at amino acids 151–155, 243–254 and 354–369. However, lumbar CSF had a different pattern still peaking at 151–155 but having low abundance towards the C-terminus. We also observed that 4 R:3 R relative abundance ratio is >2 in both, ex vivo brain and *post-mortem* profiles, suggestive of altered isoform homoeostasis.

We quantified the common post-translationally phosphorylated tau peptides in brain (Supplementary Fig. 1). We observed higher pTau208, pTau217 and pTau262 levels in samples which had evidence of amyloid plaques (AD samples) compared to samples without plaques (i.e. controls) and samples with amyloid plaques but no clinical evidence of AD, however the group differences were not statistically significant.

### Brain tau is rapidly isotopically labelled in the human brain but has a half-life of around 5 weeks

**Fig. 4:**
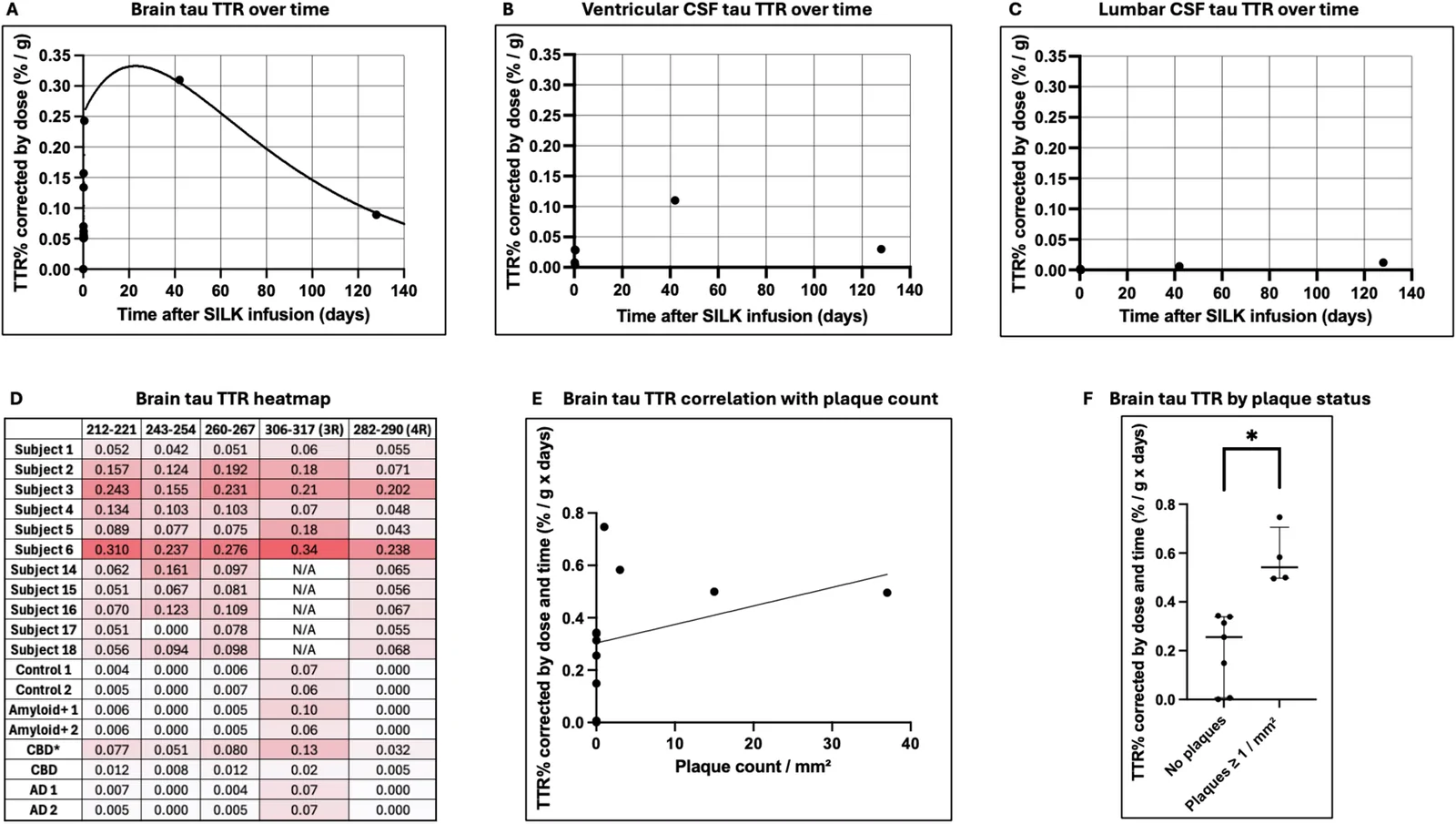
Tau stable isotope labelling results across brain and CSF. **A** Ex vivo brain tau peptide 212-221 TTR normalised to infused leucine dose (%/g) plotted against time after SILK infusion. Label is detectable by 3.28 h, with peak at day 42 (interpretation limited due to sparse timepoints). Model-derived estimate of brain tau TTR half-life is 33.6 days. **B** Ventricular CSF tau 212-221 TTR (%/g) over time shows broadly similar pattern to brain but ~3-fold lower magnitude. **C** Lumbar CSF tau 212-221 TTR (%/g) over time shows no significant labelling. **D** Heatmap of brain tau TTR normalised to dose (%/g) across peptide groups and cases. N/A indicated values not available. CBD* denotes case that received leucine infusion ~16 months prior to post-mortem; its TTR is shown without dose normalisation to avoid artefactual deflation relative to post-mortem controls without infusion. CBD is the same case corrected by dose. **E** Brain tau peptide 212–221 TTR normalised by leucine dose and sampling time (%/g × days) plotted against amyloid plaque burden. Each point represents one independent biological subject (*n* = 11). A positive association was observed but did not remain statistically significant after correction (two-sided Spearman’s correlation; Benjamini-Hochberg correction across five peptide groups; R = 0.76, adjusted *p* = 0.053). **F** Brain tau peptide 212-221 TTR normalised by leucine dose and sampling time (%/g × days) stratified by plaque status. Each point represents one independent biological subject. Samples were grouped as no plaques identified (*n* = 7 biological subjects) or plaque count ≥1 per mm² (*n* = 4 biological subjects). Plaque-positive samples showed higher tau TTR values than plaque-negative samples (two-sided Mann–Whitney U; Benjamini-Hochberg correction across five peptide groups; adjusted *p* = 0.031). Source data are provided as a Source Data file. AD Alzheimer disease, Aβ β-amyloid, CBD Corticobasal Degeneration, NPH Normal Pressure Hydrocephalus, pTau phosphorylated tau, SILK Stable Isotope Labelling Kinetics, TTR Tracer to tracee ratio.

Using IP and targeted MS, we detected incorporation of labelled leucine into tau protein in all of the ex vivo brain biopsies collected from SILK labelled individuals, and in the single labelled post-mortem disease control (Fig. 4). We demonstrated label incorporation across five leucine-containing peptides that span the proline-rich and microtubule-binding regions (MTBR) of the tau protein. Signals from SILK labelled participants were significantly higher than the natural isotopic abundance measured for each peptide transition in unlabelled controls. Figure 4A shows mean tracer to tracee ratio (TTR%) corrected by dose relative to the duration between labelling and brain biopsy. Labelling uptake occurred as early as 3 h 17 min from the start of infusion, with the highest TTR observed in the biopsy collected 42 days post label, followed by lower TTR observed 128 days post-leucine infusion. In contrast, the *post-mortem* brain (taken from Sato et al. ^31^) had much lower enrichment. The *post-mortem* tauopathy case (CBD), which was collected 16 months following label had very low TTR, demonstrating that brain leucine labelling has been mostly cleared by that point, but it still has higher than natural abundance indicating that tau can be retained in brain tissue for years (Fig. 4D). At a group level we derived the half-life of 212-221 tau peptide group in the brain to be 33.6 days (Fig. 4A). This is broadly equivalent to tau turnover in lumbar CSF^31^.

We note that the amount of labelling of the tau peptides in the brain was similar along the molecule (Fig. 4D). However, we observed a trend towards less labelling of 4 R peptide group compared to 3 R.

To determine whether brain tau synthesis rate was amyloid-dependent, we stratified ex vivo brain tau peptide 212–221 TTR (corrected by leucine dose and time) into two groups (0 plaques vs ≥1 plaque per mm^2^ identified on neuropathology). Higher tau TTR values were observed in plaque-positive samples (adjusted *p* = 0.031, Mann–Whitney U; Benjamini-Hochberg correction) (Fig. 4F). Furthermore, brain tau peptide 212-221 TTR (corrected by leucine dose and time) and the number of amyloid plaques showed positive trend but not statistically significant once adjusted for multiple comparisons (R = 0.76, adjusted *p* = 0.053, Spearman’s correlation; Benjamini-Hochberg correction) (Fig. 4E). The rest of available tau peptide TTR data showing their enrichment over time and their relationship with plaque count can be found in Supplementary Fig. 2.

### Tau in ventricular CSF is a surrogate for brain tau

To determine whether newly labelled tau was actively secreted into CSF within such a short time window, we examined the profile of tau in ventricular and lumbar CSF. The relative abundance of tau peptides in lumbar and ventricular CSF, obtained after normalisation to the mid-domain TPPS peptide, (181–190) is shown in Fig. 3A and demonstrated high concentrations of full-length tau in ventricular CSF characterised by similarly abundant tau peptides over the full tau protein sequence. Furthermore, the absolute levels of mid-domain TPPS between lumbar and ventricular samples were compared in Fig. 3B and their median difference was found to be statistically significant (*p* = 0.030). Taken together, these findings indicate that tau measured in ventricular CSF has a different profile from lumbar CSF and closely resembles the profile of tau in brain.

Tau peptide 212-221 TTR was also quantified in lumbar and ventricular CSF. Figure 4C shows tau TTR in lumbar CSF corrected by total leucine dose. There was negligible label uptake in lumbar CSF with insufficient amount of tracer administered. CSF tau SILK typically requires much higher tracer doses to observe the label at the lower tau concentration expected in CSF^31^. However, there was labelling uptake under 6 h in ventricular CSF. At a group level, the tau labelling in ventricular CSF (Fig. 4B) was similar to what was observed in brain, however TTR at similar timepoints was lower in ventricular CSF by ~3-fold and the enrichment was lower.

### Ex vivo amyloid plaques are stable structures with low turnover rates

To quantitate the translation of Aβ, we carried out IP-MS of brain homogenate across 2 centres (Gothenburg University, Sweden and Washington University, USA). We did not observe evidence of ^13^C leucine enrichment in any of the brain homogenates, using either method. Limits of detection of the IP-MS assay for Aβ1-42 at Washington University were 1.87% TTR (Supplementary Tables 3 and 4).

To further characterise and determine the maturity of plaques detected by routine pathological investigation, we quantified isomerised (Asp-1 and Asp-7 residues) species of Aβ_1-15_ in NPH brain samples. Highest amount of isomerisation (90%) was observed in *post-mortem* AD tissue, indicating the maturity of the amyloid plaques^18,44,47^. Overall, the proportion of non-native isomerised Aβ_1-15_ species in AD post-mortem and NPH samples with higher plaque load was greater (Fig. 2D). Non-native iso-Asp1/isoAsp7 and iso-Asp1,7 L were found in all NPH samples to varying degree. AD 1 and 2 had the lowest amount of native 1,7-L species (9.4–10.4%), while Subjects 3, 11 and 12 (samples with highest plaque load) had less than 40%, indicating the amyloid plaques in these NPH samples were not fully mature as observed in *post-mortem* AD (Supplementary Fig. 3).

**Fig. 5:**
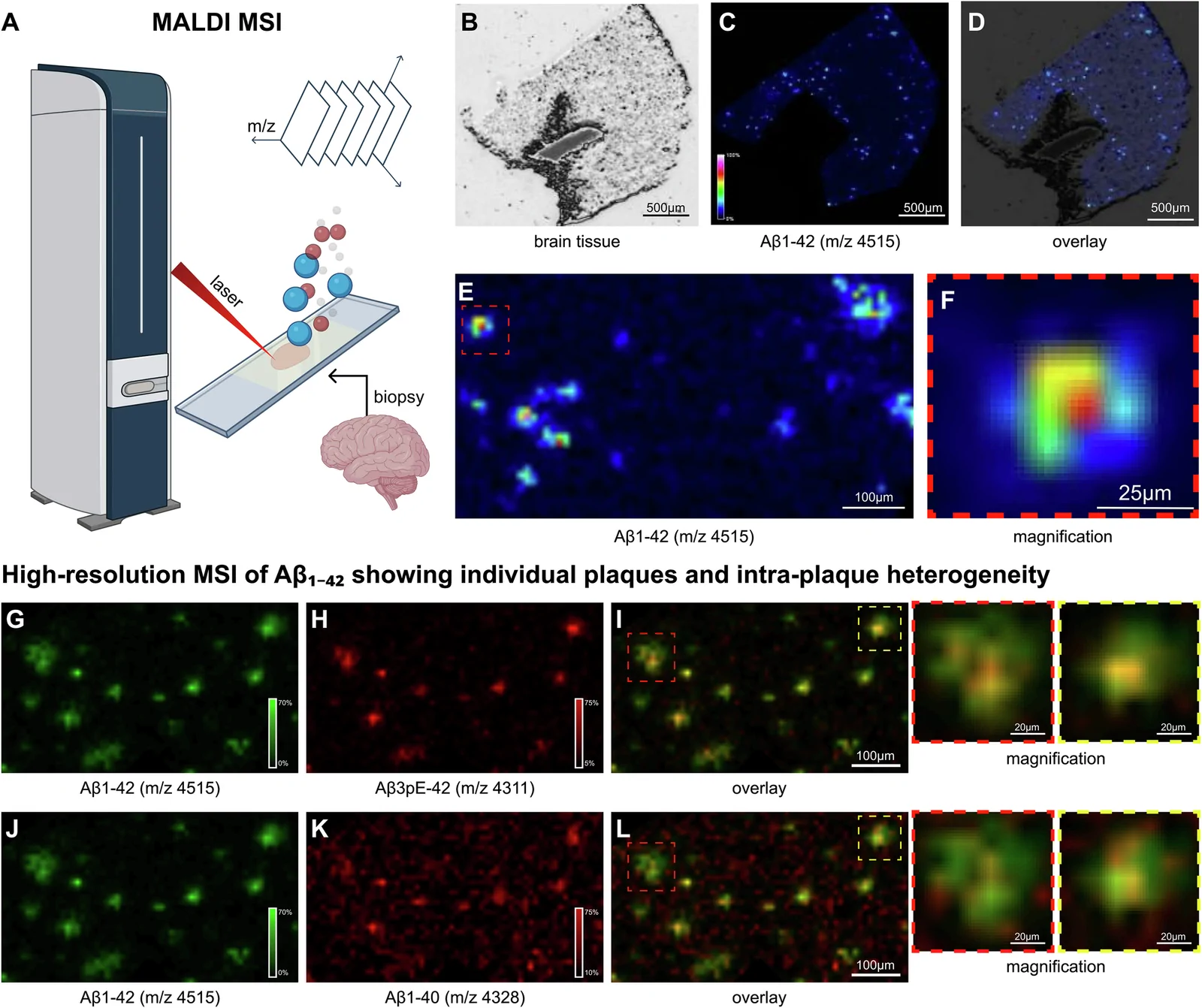
MALDI imaging of plaque pathology in the brain. **A** Schematic representation of the MALDI MSI. In situ MALDI MSI spectra are generated over a predefined x, y pixel array. Single ion images are generated by plotting ion intensities over the tissue sections. Created in BioRender. Szadziewska, A. (2026) https://BioRender.com/kjzun1z. **B** Representative optical image of a brain tissue cryosection used for MALDI MSI analysis. The MALDI MSI experiment was performed on cryosections from *n* =  13 independent subjects, with one biopsy sample available per subject and 1 tissue section analyzed per biopsy. **C** Single-ion image of Aβ1–42 (m/z 4515.4) acquired at 20 µm spatial resolution in biopsy cryosections, and **D** its overlay with the corresponding tissue morphology. **E** High-resolution MALDI-MSI (10 µm) of Aβ1–42 showing individual amyloid plaques and intra-plaque heterogeneity. **F** A representative plaque from (**E**) is shown magnified at right. Ion intensity scale: 0–100%. **G–I** Single-ion images for different Aβ species reveal distinct spatial distributions across plaques. **G** Aβ1–42 (m/z 4515.4) and **H** Aβ3pE–42 (m/z 4311.3) signals are shown individually, with **I** the overlay highlighting Aβ3pE–42 localization at the plaque core. Similarly, **J** Aβ1–42 (m/z 4515.4) and **K** Aβ1–40 (m/z 4328.0) are shown separately, with **L** the overlay and magnified insets demonstrating Aβ1–40 localization at the plaque core. Aβ β-amyloid, MALDI matrix-assisted Laser Desorption/Ionization, MSI mass spectrometry imaging, m/z mass-to-charge ratio.

**Fig. 6:**
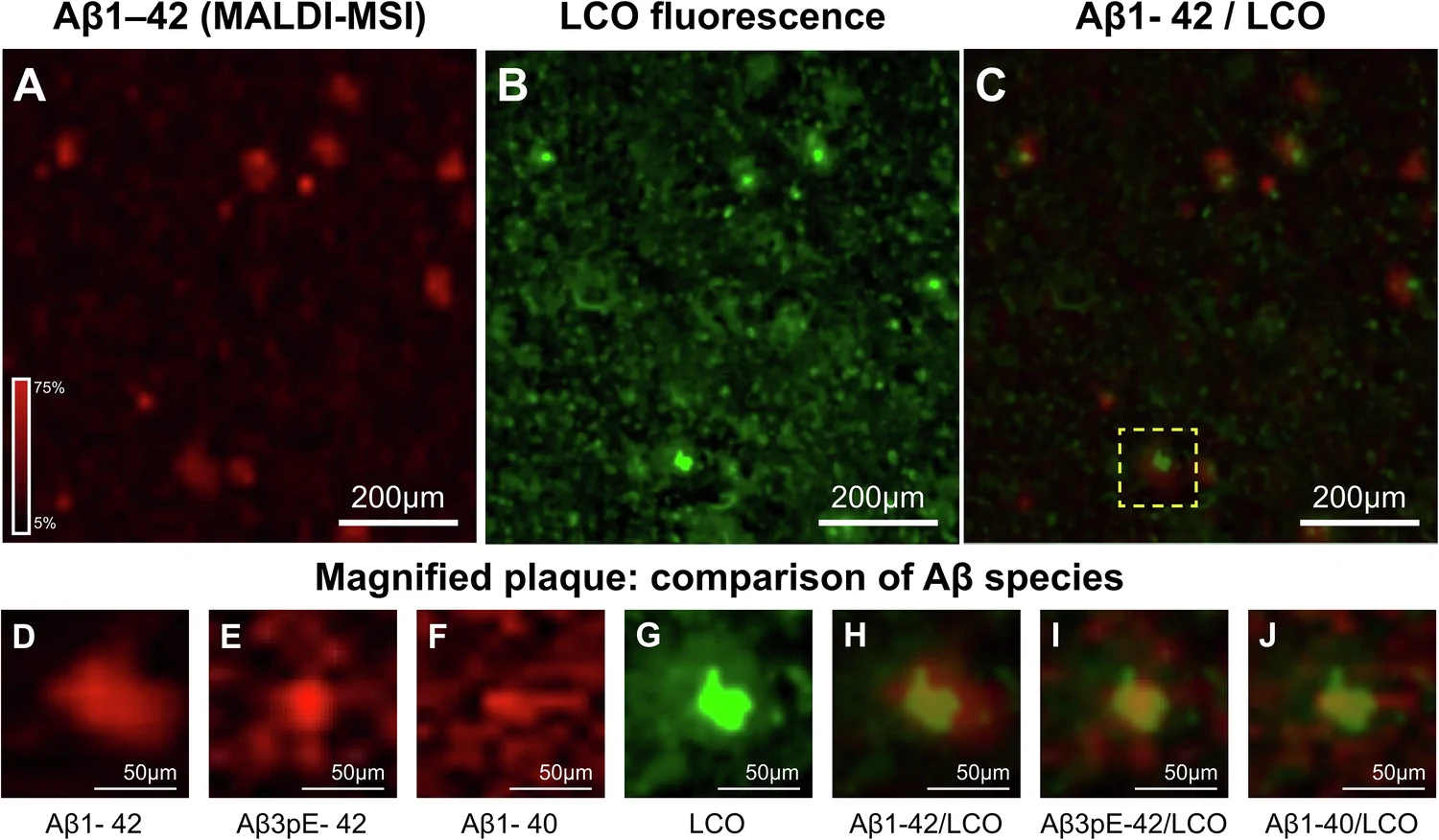
Correlative chemical imaging of plaques in the brain. **A** Single ion image of Aβ1-42 (m/z 4515.2). **B** Wide-field, fluorescence microscopy of LCO at 488 nm. The LCO staining with MALDI-MSI was performed on cryosections from *n* =  13 independent subjects, with one biopsy sample available per subject and 1 tissue section analyzed per biopsy. **C** Overlay of MSI and fluorescence channels reveals spatial correspondence between Aβ1–42 and LCO-positive plaques. **D–F** Magnified view of the boxed plaque in (**A–C**), showing individual Aβ species and their relationship to the LCO signal (**G**). Overlay of LCO and Aβ shows localization of Aβ1-42 across the entire plaque (**H**), while Aβ3pE-42 (**I**) and Aβ1-40 (**J**) localize to mature, core structure outlined by LCO intensity. Scale bars: 200 µm in panels A–C and 50 µm in panels (**D**–**J**). Aβ β-amyloid, LCO luminescent conjugated oligothiophenes, MALDI Matrix-assisted Laser Desorption/Ionization, m/z mass-to-charge ratio.

To further characterise amyloid plaque pathology in situ, we used correlative MALDI MS imaging together with functional amyloid microscopy with LCO probes^48^ as previously demonstrated in mouse models^49^ and *post-mortem* brain^50^, providing information on Aβ distribution (MSI) and plaque maturity (LCO). MALDI-MSI was performed on brain biopsy cryosections, generating spatially resolved mass spectra across the tissue. Plaque-associated MALDI mass spectra acquired directly from individual deposits confirmed the presence and relative abundance of Aβ isoforms in situ (Fig. 5A–C). The most prominent Aβ species found in single deposits included Aβ1-42, Aβ1-40, Aβ4-42, Aβ3pE-42 and Aβ11pE-42 (Supplementary Fig. 4). To resolve plaque microarchitecture, high-resolution MALDI-MSI was performed at 10 µm pixel size, revealing individual amyloid deposits with marked intra-plaque heterogeneity in Aβ1–42 signal intensity (Fig. 5E). Magnification of representative plaques demonstrated non-uniform peptide distributions within single deposits (Fig. 5F). Structural amyloid imaging provided through correlative LCO microscopy revealed differential Aβ peptide localization across and within individual plaques. Specifically, cored plaques showed characteristic localizations of Aβ1-40 to the core while Aβx-42 species including Aβ1-42 and Aβ3pE-42 showed a more homogenous distribution across both plaque core and diffuse/immature periphery (Fig. 6). Together, these findings highlight pronounced peptide-specific spatial organization within amyloid plaques.

## Discussion

In this study, we examined brain tissue from living individuals with neurodegenerative pathology and labelled pathologically relevant proteins ex vivo. We were able to capture and characterise very early brain Aβ and tau pathology and provide ex vivo evidence for the interaction between Aβ and tau by demonstrating the spatial association between tau phosphorylation and plaque deposition. We also compared the tau profiles of matched ventricular and lumbar CSF samples and the profiles directly with that in the brain, showing that ventricular CSF during shunt surgery more closely resembles brain tissue, and may serve as a surrogate for a brain biopsy. Finally, we showed that we can date AD pathological changes ex vivo, through detailed biochemical characterization and quantitation of PTMs, and by measuring the incorporation of new labelled peptides in brain during life using IP-MS and MALDI imaging. We found that tau is translated rapidly and the isotopic infusion into newly synthesized tau is measurable in soluble brain extract almost immediately. This enabled us to estimate that tau turnover in brain is ~34 days which is similar to the turnover measured in lumbar CSF; currently estimated to be 23 days^31^. Differences might have been expected as intracellular tau clearance mechanisms^51,52^ differ from extracellular tau clearance^53,54^.

As expected, ventricular CSF Aβ1-42/40 ratio had good predictive value for detecting amyloid pathology in the parietal cortex, however the rest of the pilot ventricular CSF biomarkers showed no correlation with amyloid plaques. This may be due to intraoperative ventricular CSF contamination from the brain tissue and further studies would be needed to explore this.

We gain insights into the chemical composition of amyloid plaques. As previously seen in APP mouse models and in *post-mortem* human tissue^22,55^, ex vivo plaques contain a core of Aβ1-40 and pyroglutamate modified Aβ while Aβx-42 species showed more homogenous distribution^56,57^. This substantiates earlier suggestions that pyroglutamation of Aβ underlies progression of amyloid plaques ex vivo^55^. The clinical translational importance of pyroglutamation in AD, in the context of emerging amyloid therapies, is highlighted by this data. Donanemab is a humanised immunoglobulin G1 monoclonal antibody that specifically targets N-terminally truncated pyroglutamate modified β-amyloid (AβpE) and has been shown to reduce plaque load, the rate of cognitive decline, and tau tangle burden in individuals with AD^58,59^. Its rapid clearance of cerebral amyloid as measured by Aβ PET and thereby its clinical efficacy may be due to AβpE being specifically located in cortical plaques^58–60^.

We have been able to determine plaque age based on Aβ isomerisation. Imaging and fluid biomarkers have repeatedly demonstrated Aβ accumulation begins more than 20 years before the onset of AD clinical symptoms^4,61–63^. Evidence supports that this is a result of decreased β-amyloid clearance^30,64^. The impairment in clearance increases the half-life of the β-amyloid peptides and the process of AD pathology entombs the peptide for decades making it a long-lived peptide^18^. Being able to age these peptides can be very useful in disease staging and future therapeutic decision-making. So far, the most common structural modifications associated with aging are the isomerisation of aspartate/asparagine (Asp/Asn) which have been particularly useful for protein dating^18,65–67^. Specifically, Aβ has two Asp residues (Asp-1 and Asp-7) at the N-terminus that can undergo individual isomerisation, and each peptide has a combination of their L/D and/or iso-L/iso-D forms resulting in total of sixteen Aβ_1-15_ isomers. Five of these isomers shown in Fig. 2C were the most abundant in AD and it was estimated that around 50% isomerised Aβ_1-15_ correlates with mean plaque age of 8 months, while 85% isomerisation indicates age of 4 years^18^. Our study aligns with these findings, showing that the isomers from the AD sample (which are anticipated to be the oldest due to the established disease) indeed are the oldest as they have around 90% isomerised Aβ_1-15_ while the isomers from Subjects 3, 11 and 12 contain over 60% isomerised species. These findings illustrate the utility of quantifying isomerised species in differentiating the plaques by age. Amyloid burden does not always correlate with disease severity or cognitive impairment in sporadic or familial AD^68,69^. Biochemical staging of amyloid plaques is based on the heterogeneity of Aβ isoforms and modified species that could be detected with AD patients compared to pathologically preclinical AD cases^70^. At the earliest stages Aβ_42_ aggregates to form diverse forms of plaques, with time other Aβ isoforms aggregate and accumulation of multiple PTMs on Aβ takes place^69,70^. Plaque age, and the structural maturity are crucial factors in how an initial nidus could impart neurotoxicity with time^44,71^. Most of our conventional fluid and radiological biomarkers are not designed to accurately differentiate the ‘age’ of the neurodegenerative process. This is likely to become increasingly important in therapeutic trials where many experts believe that we will have a more ‘tailored’ approach in treating patients with AD depending on the stage of its progression, highlighting the importance of accurately assessing the age of the neurodegenerative process^72^. Furthermore, we can see that the densest isoforms in the brain slices corrected by the weight across the group are Aβ_16-27_ and Aβ42 (Fig. 2B). This observation supports the previous findings in literature showing that the levels of Aβ_16-27_ are significantly higher in samples with AD^18^ and can be used in the brain as a proxy for amyloid aggregation^73^.

Overall, the profile of tau ex vivo was similar to *post-mortem* tissue. However, we noted a statistically significant difference in abundance of certain tau peptide groups in N-terminus and C-terminus in the ex vivo brain compared to *post-mortem* samples which may be suggestive of structural changes or tau truncation processes that occur *post-mortem* (Fig. 3A). It raises the possibility that *post-mortem* preparation delay and the way the samples are handled can affect the structure or integrity of proteins including tau^74–76^. Although adult brain is described to have roughly balanced 4 R and 3 R^77^, our data shows higher 4 R:3 R ratio in the soluble fraction of brain tissue ( > 2). This is consistent with altered homoeostasis which supports previous evidence for a shift towards lower 3 R abundance in prodromal/AD pathology^78^. This could be explored further to understand its implications in tauopathies where the 4 R:3 R ratios vary.

Although we found that pTau208, pTau217 and pTau262 levels were raised in AD *post-mortem* brain samples compared with other *post-mortem* controls and NPH samples, the difference was not statistically significant (Supplementary Fig. 1). Small number of samples is a major limitation and therefore more samples are needed to further comment on this.

We observed isotopic labelling, and therefore new translation, of peptides that represent the full length of the tau protein in brain, within only ~3 h, indicating that tau is rapidly translated in brain tissue, even if downstream biomarkers of tau synthesis take days to be measurable in lumbar CSF^31^. We establish that the half-life of tau in the brain tissue is ~34 days, by a third slower than in lumbar CSF when corrected by leucine dose (from Sato et al. ^31^). We are able to provide insight into the relationship between brain and CSF tau turnover, which will inform clinical trial design and inform timing of sample collection when measuring potential clinical responses to tau therapies. It is important to highlight that the tau half-life data is based on a statistical model and our data is lacking key timepoints around the peak of the curve, therefore more data will be needed to confirm our preliminary findings.

It was noted that the 4 R:3 R TTR ratios in all NPH samples were <1, suggesting a trend towards reduced appearance of newly labelled 4 R tau compared to other tau isoforms. This could suggest that this isoform has slower translation or truncation. Alternatively, clearance or incorporation into pathology may be different to other tau isoforms. While this study cannot definitively address these questions, we show that different tau isoforms can be tracked in living human brain, which could be exploited in future work.

We also observed a correlation between tau labelling and amyloid plaque density (Fig. 4E and Supplementary Fig. 2). As our study does not have the statistical power to show significance, further studies will be needed to confirm this. Furthermore, tau peptide group 212–221, which is the group that is used to measure TTR due to its relatively high abundance in CSF, shows significant difference in TTR (corrected by dose and time) between samples with plaques and no plaques (Fig. 4F). These observations provide ex vivo support to previous studies which suggest that tau production rate is increased in presence of amyloid pathology^31,32^.

We observed a tau SILK labelling pattern and tau profile in ventricular CSF during shunt surgery that reflects brain tau profile rather than lumbar CSF. This is highly likely to reflect inevitable iatrogenic release of intracellular and brain derived proteins during shunt insertion rather than reflecting physiological neuronal secretion of tau into the extracellular space and exchange into ventricular CSF. Our study design required lumbar CSF to be collected at the start of the VP shunt insertion, which meant that ventricular CSF was always collected afterwards, and following the procedure of inserting the shunt through the cortex and parietal lobe potentially introducing procedure related release of brain proteins into the ventricular CSF. We observed a small amount of tau SILK labelling in ventricular CSF, which was not seen in lumbar CSF, within ~3 h of labelling. This is not consistent with previous tau SILK data which showed that newly synthesized tau took 3 days to be released into lumbar CSF in healthy individuals and in AD^31^. Furthermore, tau that is released into lumbar CSF physiologically is truncated^31^. We observed full length tau in CSF and other tau isoforms which are not usually seen in human CSF^79^. This observation raises concerns about previous biomarker studies that demonstrate a gradient between lumbar and ventricular CSF tau concentrations^80,81^. Rather, once correcting for a peptide that represents full length tau, and therefore corrects for brain protein contamination, we found no evidence for a difference in relative abundance of tau isoforms between ventricular and lumbar CSF. Furthermore, we showed a statistically significant difference in absolute levels of the peptide used for correction when compared in ventricular and lumbar CSF which further supports our argument. This finding opens an exciting avenue of ventricular CSF acting as a surrogate for brain biopsy and this deserves to be investigated further in the future as a less invasive way of access to the brain compartment ex vivo. This is especially important for patients with NPH who are known to have higher incidence of neurodegenerative pathology, and undergoing a brain biopsy during shunt insertion is practised by many experts as a diagnostic and prognostic adjunct^82,83^. It provides a potentially valuable resource to study tau, NfL and other intracellular neuronal proteins which are found in much higher abundance within brain than CSF or plasma^84,85^. It is however important to note that we do not know whether ventricular CSF collected intraoperatively shows the true tau secretion and distribution as it may be influenced by the procedure related release of brain proteins as mentioned above. Therefore, ventricular CSF collection outside the operating theatre (e.g. shunt reservoirs) would be required to more confidently characterise ‘non-contaminated’ ventricular CSF proteins and kinetics or indeed to confirm the similarity with intraoperative ventricular CSF.

We did not see any significant tau labelling in lumbar CSF. We know from previous studies that 4 mg/kg/hr leucine for 16 h is required to achieve sufficient labelling of tau in lumbar CSF^30–32^. As our primary aim was to look at tau production and amyloid plaque turnover in brain homogenate—we did not follow the above method and gave ~2 mg/kg/hr leucine infusion instead, and for much shorter duration, dictated by clinical care. Therefore, we believe that the lack of tau label uptake in lumbar CSF was simply due to insufficient labelling.

By contrast, we did not observe Aβ labelling in insoluble amyloid plaques within the same time window. Due to limited quantity of brain tissue, we were unable to measure SILK labelling in the soluble fraction of brain homogenate, but based on previous amyloid SILK studies, where labelled soluble amyloid was observed in CSF within ~4 h, we would expect to see rapid translation of amyloid species in brain^30^. We do not think this finding is likely to be explained by technical factors. The duration of leucine infusion varied between our subjects as they were all pre-operative and the time of surgery changed due to various clinical factors. Although most of our subjects did not have a full 9 h of leucine infusion it has previously been demonstrated that the dose of leucine administered in this study is sufficient to achieve high levels of plasma leucine enrichment ( ~ 10%)^30^. We were able to establish very low limits of detection of the TTR of Aβ1-42 to demonstrate that this was not an artefact of technical limitations of our targeted mass spectrometry assay (Supplementary Table 3 and 4). We validated our findings across two independent mass spectrometry laboratories (Gothenburg University, Sweden and Washington University, USA). We posit that plaques are relatively stable structures with abundant unlabelled Aβ material, and not dynamically adsorbing new Aβ in a quantity sufficient to modify significantly the relative isotopic abundance during the few hours of the labelling process.

This study also provided insight into the spatial relationship of AD protein inclusions in biopsied brain ex vivo. Individually, Aβ and tau are implicated in the pathogenesis of AD, but exactly how they interact to cause neurodegeneration remains unknown^26–28^. The formation of dystrophic neurites around Aβ plaques is one of the key pathological hallmarks of AD pathology. These structures contain ubiquitin, neurofilament, phosphorylated tau, and APP and are associated with synaptic dysfunction^86–88^. Regional correlations between Aβ and tau have been characterised in *post-mortem* human brain^45^ and in mouse models of AD^86,89^ and most recently the presence of hyperphosphorylated tau (pTau217) has been observed in APP mice and in *post-mortem* human brains^90^. We were able to demonstrate the direct co-localization of dystrophic neurites with plaque pathology suggesting interaction and confirm ex vivo human evidence of hyperphosphorylated tau217 in these structures during life (Fig. 1). This pool of tau could be the substrate for pTau biomarkers in CSF and blood, rather than aggregated pTau from tangles which may not be easily released/secreted to CSF or plasma, and thus not available for measurement during life. This is consistent with CSF and plasma fluid biomarker data showing that phosphorylation at this site, and release of pTau217 to CSF and blood, is associated with amounts of plaque pathology^91^. It is also noteworthy that amyloid therapies^92,93^ reduce or slow the progression of tau pathology, despite having no known direct effect on tau metabolism. We directly visualise phosphorylated tau in neuritic plaques surrounding amyloid plaques, providing ex vivo evidence of the co-localisation of plaques and pTau217. AβpE has been correlated with tau phosphorylation in post-mortem human brain^43,94^ and we find intriguing support for a possible mechanistic link between amyloid pyroglutamation and tau phosphorylation.

Limitations of this study include small sample size due to clinical protocol and larger studies will be needed in the future to validate our findings. Furthermore, individuals providing ex vivo brain biopsies presented with a clinical syndrome consistent with suspected NPH rather than AD, however AD pathology is common in this population with amyloid pathology seen in around 40% of NPH cases^83^. Although we do not yet know if our subjects will develop clinically-demonstrable AD, in other longitudinal follow-up studies of NPH, those with Aβ pathology on biopsy follow a clinical course consistent with AD^95–97^. It is possible that amyloid plaque accumulation in NPH may involve altered CSF dynamics as one of the several contributing mechanisms. Impaired bulk CSF clearance is well described in NPH^53,98^ and reduced Aβ clearance has also been implicated in AD^64^. Given the overlap between these conditions^83^, altered CSF dynamics may represent one pathway that contributes to Aβ accumulation and may help explain why patients with NPH developed co-existing proteinopathies. Lastly, all our brain samples were collected from the right parieto-occipital lobe and therefore the disease burden is likely to be different in various parts of the same brain in accordance with Braak staging and this is important to note when trying to correlate biomarkers with plaque count.

In summary, we have captured very early immature amyloid plaque deposition from living human brain tissue, with high spatial and temporal resolution, with detailed biochemical characterizations of Aβ and tau species, at a stage when biomarkers are still normal or transitioning. We find that intracellular tau turnover in brain is similar to lumbar CSF tau turnover, but that certain tau isoforms may turn over at different rates which may provide insights into pathological tau aggregation. We provide detailed chemical characterization of amyloid plaques and tau ex vivo and provide immunofluorescence images of direct interaction between amyloid plaques and pTau217 in human biopsied brain tissue. We validate mouse and *post-mortem* human data demonstrating the spatial distribution of amyloid moieties in plaques, and that pyroglutamate-modified Aβ is found around plaques and is likely to be important in plaque growth as well as potentially an important target for amyloid immunotherapy. From a practical perspective, this proof of principle study shows that it is possible to capture and label neurodegenerative pathology during life, with implications for other neurodegenerative diseases beyond AD. We also provide valuable information that describes the relationship between the metabolism of key intracellular and extracellular brain proteins and their chemistry and concentration in CSF. This will inform future clinical trial design.

## Methods

This study was approved by the Bloomsbury ethics committee and all individuals provided informed written consent. This included consent for the collection and retention of brain tissue fragments released during shunt insertion and for the surgeon to obtain a brain biopsy during surgery. Participants also consented to access to relevant medical records for study monitoring and audit; sharing of study data and samples with approved research centres worldwide; retention of data and samples by UCL for 20 years and their anonymised use in future research, including research conducted by commercial healthcare companies; and necessary exchange of information between their GP and the research team. Participants understood that their participation was voluntary, that they could withdraw at any time without affecting their medical care or legal rights, and that individual research test results would not be returned. Communication between the lead author (RWP) and the MHRA in 2019 confirmed that the study was not a Clinical Trial of an Investigational Medicinal Product (IMP) as defined by the EU Directive 2001/20/EC and no submission to the Clinical Trials Unit at the MHRA was required.

### Experimental model and study participant details

We recruited participants referred to the hydrocephalus service at the National Hospital for Neurology and Neurosurgery, London, with suspected idiopathic normal pressure hydrocephalus (iNPH) undergoing VPS insertion. The schematic of NPH SILK pipeline and its linked procedures is shown in Supplementary Fig. 5. We have reported age range in order to protect participant identity given the small number of participants in this study. Sex is reported as biological sex. Participants were not financially compensated for participation.

We analysed tissue from individuals who consented to brain donation at Washington University St Louis as previously described^45^. These included individuals from Alzheimer’s Disease Research Centres (ADRC)^45^ and from the Tau TANGLES study. The Tau TANGLES study is a SILK study of individuals fulfilling clinical criteria for primary tauopathy. Individuals recruited at Washington University and UCL received an intravenous label of ^13^C_6_-leucine (4 mg/kg/hr for 16 h) with CSF collected as previously described^31^. Some individuals gave consent for brain donation, and a small number are now deceased. Demographics of individuals from ADRC and Tau TANGLES Study can be found in Supplementary Table 1.

### Autopsy procedure

Written informed consent for research use was obtained from all participants or their family members. The informed consent was approved by the Institutional Review Board of Washington University School of Medicine in St. Louis, and research was carried out according to the approved protocols and to the principles of the Helsinki Declaration.

Standard procedure for neurodegenerative brain banking was followed^99^. Briefly, participants underwent brain-only autopsy within 24 h of death (post-mortem interval (PMI) < 24 h). Following extraction of the brain and pituitary gland, each was weighed fresh. The olfactory bulbs, optic chiasm, and circle of Willis were removed. The brain, including brainstem and cerebellum, was bisected sagittally. The right hemibrain was sliced (cerebrum: coronally; brainstem: axially; cerebellum: sagittally and parasagittally), photographed, and frozen between Teflon-coated aluminium plates cooled by liquid nitrogen vapour and stored at –80̊° C for future research. The left hemibrain was fixed in formalin for a minimum of two weeks, after which the brain was processed in a standard fashion for routine neuropathological examination. Frozen sections of the right parietal lobe were used for all mass spectrometric, LCO, and other analyses requiring unfixed tissues.

### NPH SILK cohort

Age group and the sex of participants can be found in Table 1 and Supplementary Table 2. Clinical data was collected and recorded prospectively. Cognitive assessments and timed walks were carried out by physicians blinded to research data. Individuals were labelled with an intravenous ^13^C_6_-leucine obtained from CK Isotopes, UK. Leucine was dissolved in medical grade normal saline and was bolused at a rate of 3 mg/kg over 10 min and then infused at a rate of 2 mg/kg/hr using an intravenous medical pump similar to Bateman et al. protocol prior to surgery^30^. Infusion was aimed to be given for 9 h, however this was not possible for all patients as it was difficult to accurately predict the surgery time and admit patients in advance. Following administration of a general anaesthetic individuals had a lumbar puncture and 10 mL CSF was collected in a polypropylene tube. Prior to insertion of VPS, ventricular CSF as well as full thickness cortical brain biopsy was collected from the parietal lobe as described below^83^. Blood was collected simultaneously in a 10 mL EDTA tube. Blood and CSF were centrifuged at 3000 × *g* for 10 min and aliquoted as previously described^31,100^.

### Neurosurgical procedure

Individuals with probable normal pressure hydrocephalus underwent standard ventriculoperitoneal shunt insertion under general anaesthesia. Lumbar puncture was done following induction of anaesthesia in left lateral position, prior to transfer to operating table. The ventricular access point was right parieto-occipital. Burr holes were made, dura was coagulated and opened. Brain was not coagulated prior to biopsy. A small core (rice grain sized) biopsy including cortex and white matter was taken using size 15 standard surgical knife blade. The biopsy was taken from the area of brain that would have been coagulated as part of routine shunt placement. Following biopsy, any bleeding points were controlled with bipolar diathermy and ventricular catheter (Medtronic ARES antibiotic impregnated catheter) introduced directly without any use of additional cannulation. Access to cerebral ventricles was achieved in single pass in all patients. Sample was collected directly from ventricular catheter. Although exact time between catheter placement and sample taken was not measured, it is estimated to be within a couple of minutes. The rest of shunt insertion procedure was done as per routine practice. All patients underwent routine postoperative imaging, including CT scan of the brain. None of the patients had clinical or radiological adverse events from the biopsy procedure.

### Radiology reports

Brain imaging was conducted on five patients using 1.5 T or 3 T MRI scanners, with one patient undergoing a CT head scan. A consultant neuroradiologist (FC), blinded to biochemical and pathological findings, reviewed all radiological images. Brain scans were rated according to established scales^101,102^. Scheltens rating scale was used for medial temporal lobe atrophy^103^, Pasquier’s scale for global cortical atrophy^104^, Fazekas scale for white matter lesions^105^, and the idiopathic normal-pressure hydrocephalus (iNPH) Radscale^106^ was used for the radiological assessment of iNPH.

#### Biochemical CSF analysis

All biochemical tests on CSF material were performed in the Department of Psychiatry and Neurochemistry, Institute of Neuroscience and Physiology, the Sahlgrenska Academy at the University of Gothenburg, Mölndal, Sweden. Samples were distributed into 0.25–1 mL aliquots in polypropylene vials and stored at −80 °C until biochemical analyses. Core AD CSF biomarkers (Aβ1-42, Aβ1-40, T-tau and pTau181), NfL and pTau217 were measured on Quanterix (Billerica, MA, USA) HD-X instrument. Aβ1-42 and Aβ1-40 were analysed using SIMOA Neurology 4-plex-E assay kit (LOT 504153). Samples were initially diluted 30 times, however with a few concentrations measured over the last calibrator point, they were rerun with 60 times dilution. T-tau was measured using SIMOA Tau 2.0 kit (LOT 504283). Samples were diluted 30 times. NfL was measured using the SIMOA Neurology 4-plex-B assay (LOT 504050). Samples were diluted 60 times. CSF pTau217 was quantified by SIMOA ALZpath pTau217 Assay (LOT 999036). Samples were diluted 39 times. PTau181 was measured using pTau-181 Advantage V2.1 Kit (LOT 503843). Samples were diluted 30 times. All concentrations were corrected for the dilution factor^107^.

### Pathological analysis at Washington University, St Louis

Routine neuropathologic examination for diagnostic purposes was performed on fixed left hemibrains. Representative areas of the left hemibrain were stained with hematoxylin and eosin (H&E) and by immunohistochemistry (IHC) using anti-beta-amyloid (10D5, Eli Lilly), anti-phosphorylated tau (PHF1, Feinstein Institute for Medical Research, Manhasset, NY), anti-phosphorylated alpha-synuclein (pSyn, Cell Applications), and anti-phosphorylated TAR DNA-binding protein of 43 kDa (pTDP-43, Cosmo Bio USA) antibodies. All major neurodegenerative diseases were staged and reported, including Alzheimer disease neuropathologic change (ADNC); other primary tauopathies (e.g., PSP, CBD, argyrophilic grain disease, aging-related tau astrogliopathy, and others); Lewy body disease; TDP-43 proteinopathies; infarcts; vasculopathies; and other pathologies.

### Immunofluorescence and LCO staining

Immunofluorescence was employed to visualize neurofibrillary tangles and tau pathology. Frozen tissue sections were thawed at room temperature and fixed in 95% ice-cold ethanol for 10 min, followed by a 1-min rinse in 70% ethanol and a 10-min wash in 1× PBS. Sections were then blocked for 90 min in blocking buffer containing 2% bovine serum albumin (BSA), 0.1% normal goat serum (NGS), and 0.1% Triton X-100 in 0.1% PBST. Incubation was performed overnight at 4 °C using rabbit anti-phospho-Tau (pTau217) antibody diluted 1:200 in antibody diluent (0.2% PBST with 1% NGS).

Following primary incubation, sections were washed three times in 0.1% PBST (5 min each). Secondary antibody incubation was carried out at room temperature for 60 min in the dark using Alexa Fluor 647-conjugated goat anti-rabbit IgG (1:1000 in antibody diluent). After incubation, sections were washed three times for 5 min each in 1× PBS. To minimize background signal, autofluorescence was quenched using TrueBlack™ reagent according to the manufacturer’s protocol.

For visualization of amyloid pathology, LCO staining was performed. LCOs are fluorescent probes that bind selectively to β-sheet–rich protein aggregates, such as amyloid fibrils. In this study, p-FTAA (pentameric formyl thiophene acetic acid) was used to visualize amyloid pathology. Sections were incubated for 25 min with p-FTAA (1:500 in 1× PBS) in the dark, followed by a 10-min PBS wash to remove unbound dye.

To visualize nuclei, DAPI staining was performed by incubating sections with DAPI (300 nM in 1× PBS) for 2 min, followed by three 5-min washes. Slides were mounted with DAKO Fluorescent Mounting Medium and coverslipped.

### Microscopy

Multichannel fluorescence imaging of immunostained human brain sections was conducted using an automated widefield microscope (Axio Observer Z1, Zeiss, Germany). Images were acquired with a Plan-Apochromat 20×/0.8 DIC air objective. Channel-specific fluorescence was recorded using DAPI, Alexa Fluor 488, Alexa Fluor 594, and Alexa Fluor 647 filter sets. Large tiled image scans were captured and automatically stitched using the microscope’s acquisition software.

### Pathology quantification

Quantitative analysis of stained sections was performed using QuPath version 0.6.1. Tiled fluorescence images were processed using threshold-based segmentation to define regions of interest.

For tau quantification, the tissue area was manually annotated, and a visual threshold was applied to identify pTau217-positive signal. The proportion of tau-positive area was calculated as the percentage of total tissue area within the section, enabling assessment of tau pathology including neuritic threads, which were more prevalent than mature neurofibrillary tangles in these samples.

Tau accumulation within and surrounding neuritic plaques was assessed by defining concentric regions of interest corresponding to the plaque area itself and zones extending 10, 20 and 30 µm from the plaque boundary. Quantification was performed only for plaques exhibiting pTau217 immunoreactivity (*n* = 3, *N* = 12).

Amyloid plaques were identified using thresholding of the LCO (p-FTAA) signal. Detected objects smaller than 20 µm² were excluded from analysis. Plaque density was expressed as the number of plaques per mm² of tissue area. The antibodies, reagents and equipment used are listed in Table 2.

**Table 2:** Reagents and equipment used for immunofluorescence staining and microscopy

Reagent	Source	Catalogue number
anti-amyloidβ 1-16 (6E10)	BioLegend	Catalogue # 803003
Alexa-fluor488	Invitrogen Thermo Fisher Scientific	Catalogue # A32723
AT8	Invitrogen, Thermo Fisher Scientific	Catalogue # MN1020
pTau217	Invitrogen, Thermo Fisher Scientific	Catalogue # 44-744
Alexa-fluor594	Invitrogen Thermo Fisher Scientific	Catalogue # A32740
Alexa-fluor647	Invitrogen Thermo Fisher Scientific	Catalogue # A3278
Reagents		
TrueBlack™	Biotium	Catalogue # 23007
Normal Goat Serum (NGS)	Sigma Aldrich	Catalogue # G6767-100ML
Bovine Serum Albumin (BSA)	Sigma Aldrich	Catalogue # A7906-100G
DAPI	Invitrogen, Thermo Fisher Scientific	Catalogue # D1306
Other		
Zeiss Axio Observer Z1 Widefield Microscope	Zeiss	Zeiss Axio Observer Z1

Antibodies, reagents and equipment are listed with their manufacturer and catalogue number.

### Mass spectrometry methods at Washington University, St Louis

#### Brain tissue homogenization

Brain homogenization buffer (1% IGEPAL, protease inhibitors, phosphatase inhibitors in 1X PBS pH 7.4, 5 mM guanidine) was added to each brain tissue at a concentration of 0.1 mg/µL of brain tissue at 4 °C, and tissue was sonicated (Fisherbrand sonicator) for 15 s with 1 s on and 1 s off pulses using 35 % intensity. Once the samples were homogenized, they were centrifuged at 11,000 × *g* for 20 min at 4 °C, to form a supernatant and a pellet. The supernatant was aliquoted into new tubes and kept at −80 °C until use. The residual pellet was incubated in 99% formic acid for 2 h at room temperature, flash frozen in liquid N_2_ and stored at –80 °C until use.

#### Soluble tau quantitation using IP-MS

For the soluble tau analysis, we immunoprecipitated (IP) tau using tau1, HJ8.5 and HJ8.7 antibody mixture as previously described with adjustments^45,79^. To each soluble brain supernatant (30 µL, ~3 mg brain tissue), 0.01% HSA solution was added along with ^15^N 2N4R (2.5 ng, gift from Dr. Guy Lippens, France) and ^15^N tau 0N3R (2.5 ng, Promise Proteomics, Grenoble, France). Tau from each sample was immunoprecipitated using 20 µL of a cocktail of conjugated beads (mix of 50 % slurry of each tau antibody conjugated sepharose beads containing 3 µg antibody/mg beads) by incubating them with rotation overnight at 4 °C. The following day the samples were washed three times with 25 mM TEABC buffer. The proteins were then eluted off the sepharose beads using 100 % FA and the eluent was lyophilized. The lyophilized samples were resuspended in 25 mM TEABC, reduced using 5 µL of 100 mM DTT for 30 min at 65 °C. The reduced sample was alkylated using 5 µL of 100 mM IAA for 45 min in the dark at room temperature. The proteins were digested overnight using 0.4 µg trypsin at 37 °C. The digested samples (soluble tau) were desalted using the Oasis µElution HLB plate (Waters) according to manufacturer’s protocol. The eluent was lyophilized and reconstituted with 25 µL of 2 % ACN, 0.1 % FA in water prior to MS analysis on Vanquish Neo UHPLC (Thermo Scientific) coupled to Orbitrap Exploris 480. Eighteen brain tau peptides were quantified by comparison with the corresponding isotopomer signals from the ^15^N internal standard using Skyline software (version 20.2 MacCoss Lab, Department of Genome Sciences, University of Washington). The tracer to tracee (TTR %) ratios for five leucine-containing tau peptides were calculated by comparing the integrated peak area of the ^13^C_6_-leucine labelled tau peptides to the corresponding endogenous (^12^C_6_) tau peptide from each brain sample. The tau peptides monitored by mass spectrometry are listed in Table 3.

**Table 3:** Description of the tau peptides and the corresponding ^13^C_6_-leucine peptide monitored by mass spectrometry

Peptide	Domain	Residue	^13^C_6_-leucine labelled peptide
QEFEVMEDHAGTYGLGDR	N-terminal	6–23	N/A
DQGGYTMHQDQEGDTDAGLK	N-terminal	25–44	N/A
ESPLQTPTEDGSEEPGSETSDAK	N-terminal	45–67	N/A
STPTAEDVTAPLVDEGAPGK	N-terminal	68–87	N/A
QAAAQPHTEIPEGTTAEEAGIGDTPSLEDEAAGHVTQAR	N-terminal	88–126	N/A
IATPR	mid-domain	151–155	N/A
TPPSSGEPPK	mid-domain	181–190	N/A
SGYSSPGSPGTPGSR	mid-domain	195–209	N/A
TPSL(*)PTPPTR	mid-domain	212–221	mono-labelled
VAVVR	mid-domain	226–230	N/A
LQTAPVPMPDL(*)K	MTBR	243–254	mono-labelled
IGSTENL(*)K	R1	260–267	mono-labelled
VQIINK	R2	275–280	N/A
LDL(*)SNVQSK	mid-R2	282–290	mono-labelled
VQIVYKPVDL(*)SK	3 R isoform	306–317	mono-labelled
IGSLDNITHVPGGGNK	R4	354–369	N/A
TDHGAEIVYK	C-terminal	386–395	N/A
SPVVSGDTSPR	C-terminal	396–406	N/A

MTBR = microtubule binding region; (*), ^13^C_6_-leucine labelled peptide that was monitored along with endogenous as well as the ^15^N internal standard peptide. N/A, not applicable.

#### Insoluble Aβ quantitation and data analysis

For the insoluble Aβ analysis, we used HJ5.1 antibody for immunoprecipitation (IP) of Aβ as previously described^47^. Each brain pellet was reconstituted in 1.5 M Tris-HCl, pH 8.8 buffer and diluted to 500 µL using 0.01 % HSA in water. 20 µL of recombinant ^15^NAβ internal standard (0.1 ng/µL Aβ1-42, 0.01 ng/µL Aβ1-40, 0.01 ng/µL Aβ1-43 and 0.01 ng/µL Aβ1-38 prepared in 0.1 % NH_4_OH/20 % ACN) was spiked into the samples. 50 µL of HJ5.1 coupled DynaBeads (25 µg antibody/mg beads per IP) and 25 µL of 1% IGEPAL, 1X protease inhibitor, 5 mM guanidine in PBS pH 7.4 were added to the samples. The IP and bead washing steps were performed on a KingFisher automated format; Aβ peptides were eluted off beads using neat FA (99 % FA) and lyophilized to dryness. The samples were resuspended in 80 µL of 100 mM TEABC and 20 µL of Lys-N enzyme (2.5 ng/µL) was added for overnight digestion at room temperature. The following day the samples were eluted using Oasis HLB µElution plate using the manufacturer’s protocol and lyophilized. During the desalting process, 10 fmol each of AQUA internal-standard peptide for Aβ(1–15), Aβ(2–15) and Aβ(3pGlu-15) was spiked for differential quantification. The peptides were incubated in 3 % H_2_O_2_, 3 % FA for 18 h at 4 °C to oxidize the Aβ peptides containing methionine. The following day samples were desalted using an Oasis µElution HLB plate using the manufacturer’s protocol and lyophilized. The Aβ peptides were reconstituted in 2%ACN/ 2%FA solution containing 20 fmol/µL BSA tryptic peptides for MS analysis on Vanquish Neo UHPLC (Thermo Scientific) coupled to Orbitrap Exploris 480. The raw data was imported into Skyline (v4.2) with formula annotations of the targeted peptides. Eight Aβ peptides (Aβ_1-15_, Aβ_2-15_, Aβ_3pGlu-15_, Aβ_16-27_, Aβ_28-42_, Aβ_28-40_, Aβ_28-38_ and Aβ_28-43_) were quantified by comparison with the corresponding isotopomer signals from their respective ^15^N internal standard (Table 4). The stereoisomers of Aβ_1-15_ (that includes Asp-1 and Asp-7 residue) (Table 5) were quantified as previously described^18,47,108^. Briefly, Aβ_1-15_ isomerised species eluted at different retention times on the liquid chromatogram compared to the non-isomerised native species. Each individual isomerised species of Aβ_1-15_ was quantified by comparing the endogenous ^14^N peak area with the ^15^N Aβ_1-15_ internal standard (non-isomerised Asp-L species). The fraction of isomerised species of Aβ_1-15_ was calculated by dividing individual isomer by the total Aβ_1-15_ in each sample.

**Table 4:** Description of the Aβ peptides quantified using mass spectrometry

Peptide	Domain	Residue	^13^C_6_-leucine labelled peptide
DAEFRHDSGYEVHHQ	N-terminal	1–15	N/A
AEFRHDSGYEVHHQ	N-terminal	2–15	N/A
pEFRHDSGYEVHHQ	N-terminal	3pGlu-15	N/A
KL(*)VFFAEDVGSN	mid-domain	16–27	mono-labelled
KGAIIGL(*)MVGG	C-terminus	28–38	mono-labelled
KGAIIGL(*)MVGGGVV	C-terminus	28–40	mono-labelled
KGAIIGL(*)MVGGVVIA	C-terminus	28–42	mono-labelled
KGAIIGL(*)MVGGVVIAT	C-terminus	28–43	mono-labelled

(*), ^13^C_6_-leucine labelled peptide that was monitored along with endogenous as well as the ^15^N internal standard peptide. N/A, not applicable.

**Table 5:** Description of the isomerised Aβ_1-15_ peptides quantified using LC-MS

Peptide	Residue	Isomerisation on Asp	Isomer
DAEFRHDSGYEVHHQ	1-15	1,7-L	Native
dAEFRHDSGYEVHHQ	1-15	1-iso-L, 7-L	Mono-isoL
DAEFRHdSGYEVHHQ	1-15	1-L,7-iso-L	Mono-isoL
dAEFRHdSGYEVHHQ	1-15	1,7-iso-L	Di-iso-L
(d)AEFRHdSGYEVHHQ	1-15	1-iso-D,7-iso-L	Mono-D, di-iso
dAEFRH(d)SGYEVHHQ	1-15	1-iso-L,7-iso-D	Mono-D, di-iso
(d)AEFRH(d)SGYEVHHQ	1-15	1,7-iso-D	di-iso-D

Endogenous isomerised species was monitored along with the Aβ_1-15_^15^N internal standard peptide. D, Asp-L; d, Asp-iso-L; (d), Asp-iso-D.

To estimate the limit of detection and limit of quantitation of ^13^C-leucine labelled Aβ_28-42_ and Aβ_16-27_ peptide, known amount of ^13^C_6_-leucine labelled Aβ_1-42_ internal standard was spiked into insoluble AD brain formic acid (FA) extract. This sample was further serially diluted into the same AD FA extract to keep the endogenous ^12^C Aβ signal constant. These serially diluted samples were then processed with HJ5.1 antibody for IP of Aβ as described above. The data was processed using Skyline (v4.2) and ^13^C_6_-leucine labelled Aβ_28-42_ and Aβ_16-27_ peak area from the extracted ion chromatograms were compared with their respective endogenous ^12^C peak area. The limit of detection (LOD) and limit of quantitation (LOQ) were 3 times the standard deviation of the blank and 10 times the standard deviation of the blank, respectively.

#### IP-MS analysis of CSF Tau profile, pTau, and Tau212-221 tracer to tracee (TTR %) ratios

CSF tau profile, pTau concentrations and corresponding phosphorylation occupancies, and tau212-221 TTR% were measured at the department of Neurology, Washington University School of Medicine (St. Louis, MO, USA) using three different IP-MS assays.

##### CSF tau profile

In this protocol, 100 µL samples were spiked with 2.5 ng ^15^N labelled recombinant 2N4R Tau (gift from Dr. Guy Lippens, France) and master mix containing detergent and chaotropic reagents (final concentration 1% IGEPAL, 1X protease inhibitor, 5 mM guanidine), and the samples were immunoprecipitated with Tau1, HJ 8.5, and HJ 8.7 conjugated sepharose beads for 2 h at room temperature. The beads with tau precipitate were washed with 25 mM triethyl ammonium bicarbonate buffer (TEABC) three times then digested on-beads with 400 ng MS grade trypsin for 16 h at 37 °C. Digested peptides were spiked with AQUA internal-standard peptides for differential quantification and then proceeded with an oxidation step with H_2_O_2_/FA solution (3%/3%) overnight. Oxidized digestion was extracted on SEP PEK μ-elution plate (Waters); the eluate was dried, kept at –80 °C, and resuspended prior to MS analysis.

##### CSF pTau concentrations and corresponding phosphorylation occupancy measurement

450 µL CSF was spiked with 2.5 ng uniformly labelled ^15^N 2N4R Tau internal standard and master mix as described above, and the samples were immunoprecipitated with Tau 1, HJ 8.5, HJ 8.7, and HJ 34.8 conjugated sepharose beads for 2 h at room temperature. Beads were washed three times as mentioned above and digested by 400 ng trypsin overnight at 37 °C. Digested peptides were extracted and cleaned by SEP PEK μ-elution plate (Waters); eluate was dried and stored in –80 °C, resuspended prior to MS analysis.

##### CSF Tau212-221 tracer to tracee (TTR %) ratios

A separate aliquot of 900 µL CSF, spiked with 2.5 ng of uniformly labelled ^15^N 2N4R Tau internal standard and master mix (final 1% IGEPAL, 1X protease inhibitor, 5 mM guanidine), was immunoprecipitated with Tau1 conjugated sepharose beads for 2 h at room temperature, following which the beads were washed three times as mentioned above and digested by 400 ng trypsin overnight at 37 °C. Digested peptides were extracted as described above using SEP PEK μ-elution plate (Waters); eluate was dried then resuspended prior to MS analysis.

#### Mass spectrometry

The peptides were directly loaded onto HSS T3 75 μm × 100 μm, 1.8 μm C18 column (Waters) heated to 65 °C using a Vanquish Neo UHPLC (Thermo Fisher Scientific, San Jose, CA, USA) and peptides were separated using a flow rate of 0.4 µL/min using buffer A (0.1% FA in water) and buffer B (0.1% FA in acetonitrile). Tau peptides were eluted from the column with a gradient of 4–8% of buffer B for 10 min, then 8-20% of buffer B for another 8 min before ramping up to 95% buffer B in next 2 min and cleaning the column for another 2 min. The Aβ peptides were eluted from the column with a gradient of 2–15% of buffer B for 10 min, 15–35% for another 10 min before ramping to 85% of buffer B and cleaning the column for the next 2 min. The Orbitrap Exploris 480 was equipped with a Nanospray Flex electrospray ion source (Thermo Fisher Scientific, San Jose, CA, USA) and operated in positive ion mode. Peptide ions sprayed from a 10  μm SilicaTip emitter (New Objective, Woburn, MA, USA) into the ion source (spray voltage = 2200 V), were targeted and isolated in the quadrupole. Isolated ions were fragmented by HCD and ion fragments were detected in the Orbitrap (resolution of 15,000 for tau and 60,000 for Aβ peptides, mass range 150–1500 m/z).

### Mass spectrometry methods at Gothenburg University

#### Protein extraction from brain biopsies and immunoprecipitation of Aβ species

Frozen brain samples were homogenized according to an established protocol^109^ with minor modifications, to get a soluble and an insoluble fraction. Brain biopsies were initially homogenized in (Tris)-buffered saline (TBS), pH 7.6 containing protease inhibitor (complete Protease Inhibitor tablets, Roche), twice for 2 min at 30 Hz using a TissueLyzer II (Qiagen). After centrifugation (30,000 x *g* for 1 h at 4 °C), the supernatant (TBS soluble fraction) was stored at –80 °C. The pellet was resuspended and homogenized in 1 ml of 70% (v/v) formic acid (FA). After sonication for 30 s and further centrifugation at 30,000 x *g* for 1 h at 4 °C, the supernatant (FA insoluble fraction) was dried in a vacuum centrifuge. Aβ peptides were immunoprecipitated from both fractions using a combination of two mouse monoclonal antibodies, 6E10 and 4G8 (BioLegend), respectively binding to the N- and mid-terminal part of the Aβ peptide. Four µg of each antibody was independently conjugated with 50 µl IgG-coated magnetic beads (Dynabeads M-280 Sheep anti-mouse, Thermo Fisher Scientific) according to manufacturer’s instructions. After conjugation with the beads, the two antibodies were combined and added to each sample. Prior to immunoprecipitation (IP), the dried FA fraction was reconstituted in 200 µL 70% FA (v/v) and shaken for 30 min at room temperature. The reconstituted samples were then moved to a new tube and the acidic pH was neutralized with 0.5 M Tris buffer till neutrality. TBS and FA-reconstituted fractions were incubated overnight at + 4 °C with antibody-conjugated beads in 0.2% Triton X-100 solution. Given the limited amount of initial material, the whole brain extract was used for IP. Two samples of CSF pool were immunoprecipitated at the same time and used as positive controls. The day after, samples underwent a series of washes and Aβ peptides were eluted in 100 µl 0.5% FA. Eluates were dried down in a vacuum centrifuge and stored at −80 °C pending MS analysis.

#### Mass spectrometry analysis of the brain extracts

Aβ peptides immunoprecipitated from TBS and FA fractions were analysed by matrix-assisted-laser-desorption/ionization time-of-flight/time-of-flight MS (MALDI-TOF/TOF-MS) and by nanoflow liquid chromatography (LC) coupled to electrospray ionization (ESI) hybrid quadrupole–orbitrap tandem MS (Dionex Ultimate 3000 system and Q Exactive, both Thermo Fisher Scientific). Prior to MS analysis, samples were reconstituted in 5 μl 0.1% FA:20% acetonitrile in water (v/v/v), shaking for 30 min at room temperature. Two of the 5 μl were used for MALDI analysis. Analysis was performed using a Bruker Daltonics UltraFleXtreme instrument in reflector mode. MALDI samples were prepared using the seed layer method and seeded in duplicate as previously described^110^. An average of 5000 shots were acquired for each spectrum (1000 at the time using a random walk mode), with 75% laser power and 5000 Hz laser frequency, and the MS thin layer method. For a more detailed analysis, the 3 μl left from MALDI preparation were dried and reconstituted in 7 μl 8% FA:8% acetonitrile in water (v/v/v) and analysed by LC-MS/MS as described previously^111^. Database search (including isotope and charge deconvolution) was performed with PEAKS Studio Xpro (Bioinformatics Solutions Inc.) against a custom-made APP database.

#### Mass spectrometry imaging analysis

*Frozen* brain biopsies were cut into 12 μm thick sections using a cryostat microtome (Leica CM 1950, Leica Biosystems, Nussloch, Germany) at –18 °C, collected on indium tin oxide (ITO) conductive glass slides (Bruker Daltonics, Bremen, Germany), and stored at –80 °C. Prior to analysis, tissue sections were thawed under vacuum for 30 min.

Prior to matrix application, defrosted tissues were treated by sequential washes of 95% EtOH for 60 s, 70% EtOH for 30 s, Carnoy’s solvent (60% EtOH, 30% chloroform, 10% acetic acid) for 90 s, 95% EtOH for 10 s, H_2_O with 0.2% TFA for 60 s, 95% EtOH for 15 s. For Aβ peptide signal enhancement, the tissue sections were hydrolyzed by exposure to vapour of concentrated formic acid for 25 min. 2,5-dihydroxyacetophenone (2,5-DHAP) matrix compound was applied using a HTX TM-Sprayer (HTX Technologies, Carrboro, NC, USA) combined with an HPLC pump (Dionex P-580, Sunnyvale, CA, USA).

MALDI MSI was performed on a Bruker rapifleX TissueTyper TOF/TOF mass spectrometer (Bruker Daltonics) instrument using the flexImaging software (v.5.1, Bruker). Peptide MSI data was acquired at 10 and 20 μm spatial resolution, at a laser pulse frequency of 10 kHz with 200 shots collected per pixel. Data was acquired in linear positive mode (LP) (10 μm) and reflector positive mode (RP) (20 μm) in the mass range of 1500–6000 Da. Pre-acquisition calibration of the system was performed using a combination of peptide calibration standard II and protein calibration standard I, to ensure calibration over the entire range of potential Aβ species.

### Statistics and reproducibility

The overview of sample availability across the NPH and *post-mortem* cohorts is shown in Supplementary Table 5. Not all the samples were included in every experimental component of the study due to limited sample availability and resource considerations.

No statistical method was used to predetermine sample size.

The mean value was presented for duplicate results unless one of the values was 0 in which case the other value was taken instead as the former result was likely due to technical error.

The experiments were not randomized.

Apart from the neuroradiologist (FC) who was blinded to biochemical and pathological findings, the rest of the investigators were not blinded to outcome assessment.

Due to small number of samples, the data was treated as non-parametric. Statistical difference between two groups was assessed using two-sided Mann-Whitney U test (for 2 groups) and Kruskal-Wallis test (for more than 2 groups). Statistical correlation was assessed using Spearman’s test. Benjamini-Hochberg correction was used for multiple comparisons. GraphPad Prism was used for all statistical analysis.

The TTR data points were normalized by the dose of leucine in grams to account for differences in labelling period length. Tau 212-221 peptide was used for modelling as it provided the strongest and most consistent signal. Leucine labelling data was incomplete, so the leucine time course from over 100 human subjects was used as a surrogate. In this cohort, the mean plateau TTR of plasma leucine was 0.4199 during the 16-h labelling period (4–16 h). This value was used as the plateau for this simulation. Because labelled leucine infusion was halted just prior to surgery for shunt placement, the total dose varied considerably. The measured tau TTR was thus normalized by the dose (i.e. TTR/g). To harmonize the model to this value, the length of labelling was set such that a hypothetical subject that was 80 kg would receive 1 g ^13^C_6_-Leu, which corresponds to 0.25 days of labelling. Subject weights ranged from 70 to 112 kg with a median of 86.4 kg. Following cessation of the hypothetical 6 h infusion, the plasma leucine TTR was modelled as a triple exponential with the formula: Plasma leucine TTR = 0.4199*(0.3022*EXP(-38.32*(time–0.25)) + 0.3022*EXP(–6.313*(time–0.25)) + 0.0321*EXP(-0.0294*(time–0.25))) with time in days. A one compartment model was used to fit the tau labelling data with a single scaling factor, with the leucine data as the input to the model, thus two parameters were fit with rate constant = 0.02064 and scaling factor 0.48. Modelling was performed in SAAM-II version 2.3.3 (Nanomath, Spokane, WA).

### Reporting summary

Further information on research design is available in the Nature Portfolio Reporting Summary linked to this article.

## Supplementary information

Supplementary Information

Transparent Peer Review file

Reporting Summary

## Source data

Source Data

## Data Availability

Source data are provided with this paper. Any additional raw data supporting the findings of this study, with participant data anonymised where applicable, are available from the corresponding authors.
