# Supplementary material for: Spatiotemporal characterization of Alzheimer disease pathology in living human brain tissue
Source: Nat Commun. 2026 Sep 24;17:9878. doi: 10.1038/s41467-026-76494-4 (PMC13612511; doi:10.1038/s41467-026-76494-4)
Supplement: Supplementary file 1 [file 41467_2026_76494_MOESM1_ESM.pdf]

Supplementary Figures

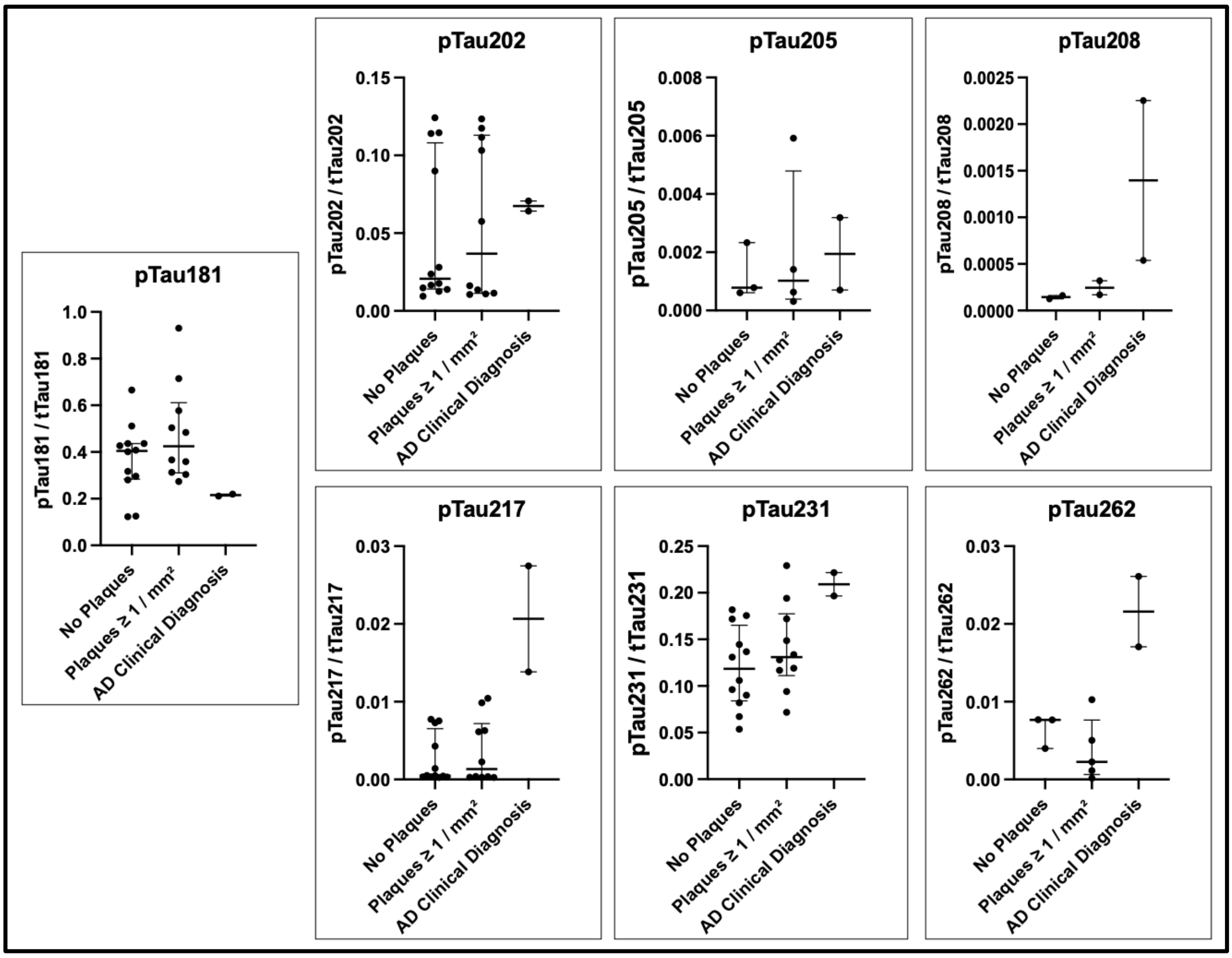

Supplementary Figure 1. Ratios of phosphorylated tau epitopes to total tau in ex vivo brain tissue

Ratios of pTau181, pTau202, pTau205, pTau208, pTau217, pTau231 and pTau262 to their corresponding total tau measures are shown across plaque status groups (No plaques; Plaque count ≥ 1 mm^2 and a clinically diagnosed post-mortem AD group). Each dot represents an individual sample; horizontal bars and whiskers indicate median and IQR. There was no visual difference in levels between samples with no plaques and samples with plaques with no diagnosis of AD. Although pTau208, pTau217 and pTau262 levels appear higher in the AD group compared with the other two groups, the group differences were not statistically significant using Kruskal-Wallis test.

Source data including the sample sizes and p values are provided as a Source Data file.

Abbreviations: AD, Alzheimer disease; Aβ, β-Amyloid; CBD, Corticobasal Degeneration; IQR, Interquartile Range; pTau, phosphorylated Tau; tTau, total Tau.

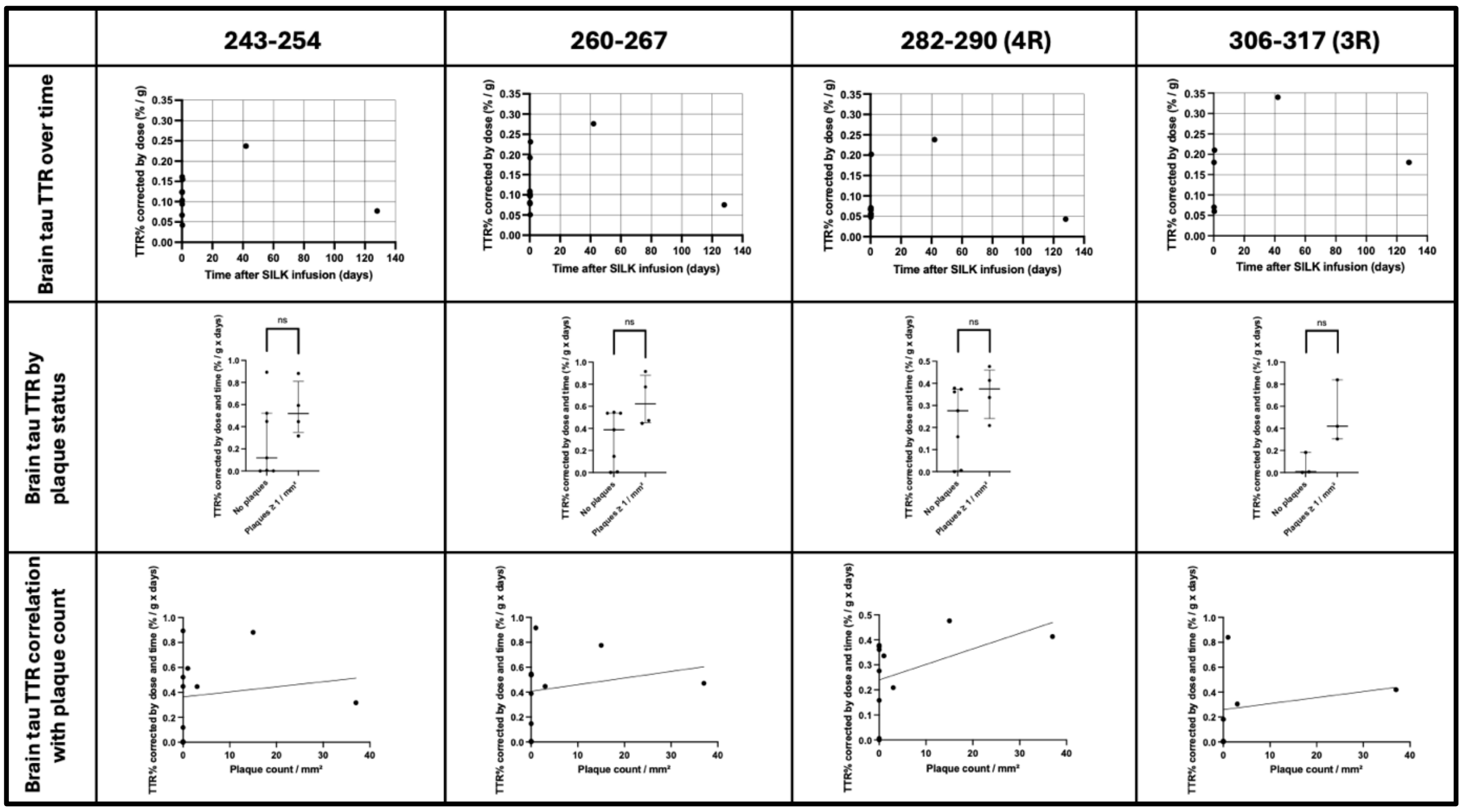

Supplementary Figure 2. Ex vivo brain tau TTR across multiple peptides

Tau TTR was quantified in ex vivo brain tissue for peptides 243-254, 260-267, 282-290 (4R) and 306-317 (3R).

Top row: Ex vivo brain tau TTR normalised by leucine dose (% / g) plotted against time after SILK infusion; each dot represents one sample. 306-317 (3R) shows the highest TTR peaking at day 42.

Middle row: Ex vivo brain tau TTR normalised by leucine dose and time (% / g x days) stratified by plaque status (No plaques vs Plaques ≥ 1 mm^2). Points represent individual samples, horizontal line and whiskers indicate median and IQR. No statistically significant differences were detected between plaque status groups for any peptide (Mann-Whitney U test).

Bottom row: Ex vivo brain tau TTR normalised by leucine dose and time (% / g x days) plotted against plaque count per 1 mm^2. No statistically significant correlations were observed (Spearman) although weak positive trends are visually apparent.

Source data including the sample sizes and p values are provided as a Source Data file.

Abbreviations: IQR, Interquartile range; TTR, Tracer to tracee ratio

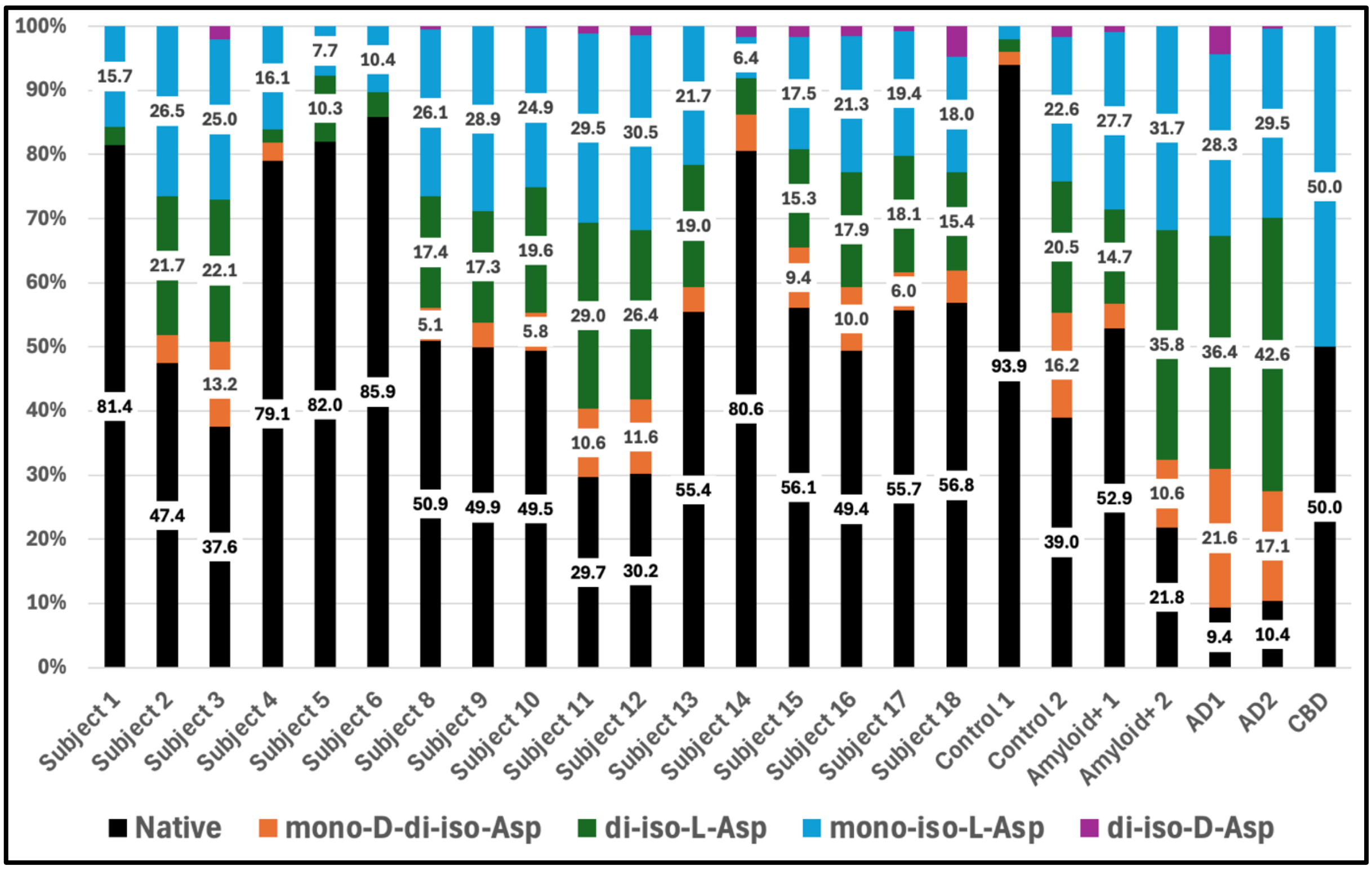

Supplementary Figure 3. Proportions of $A\beta_{1\text{-}15}$ Asp1,7 isomerisation states in NPH brain biopsies and *post-mortem* controls

Higher fraction of non-native isomers is observed in NPH cases with higher plaque density (e.g. Subjects 3, 11, 12) and in plaque-positive *post-mortem* controls (AD1, AD2, Amyloid+2).

Abbreviations: AD, Alzheimer disease; Amyloid+, Amyloid positive; CBD, Corticobasal Degeneration.

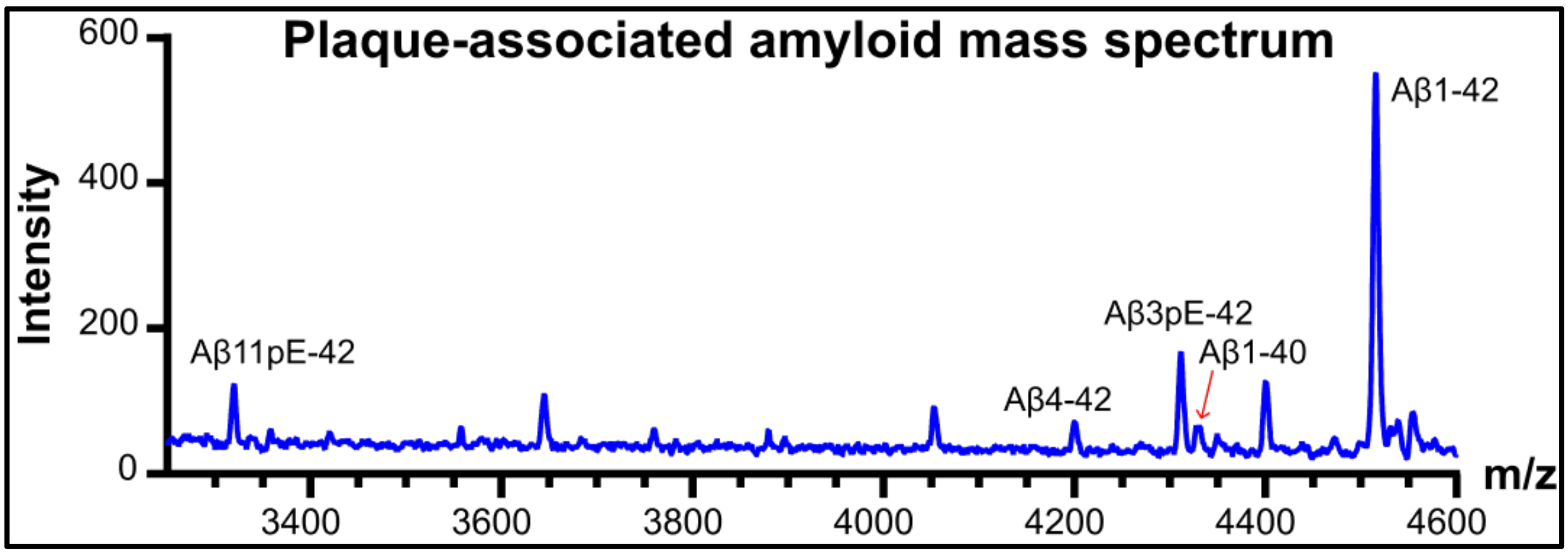

Supplementary Figure 4. Plaque-associated amyloid mass spectrum.

Representative MALDI mass spectrum acquired from an individual amyloid plaque, illustrating the relative abundance of major Aβ peptide species detected in situ. Prominent peaks correspond to full-length and N-terminally truncated Aβ isoforms, including Aβ1-42, Aβ1-40, Aβ4-42, Aβ3pE-42, and Aβ11pE-42, labelled according to their respective m/z values.

Abbreviations: Aβ, β-amyloid peptide; MALDI, matrix-assisted laser desorption/ionization; MS, mass spectrometry; m/z, mass-to-charge ratio; MSI, mass spectrometry imaging; pE, pyroglutamate-modified N terminus.

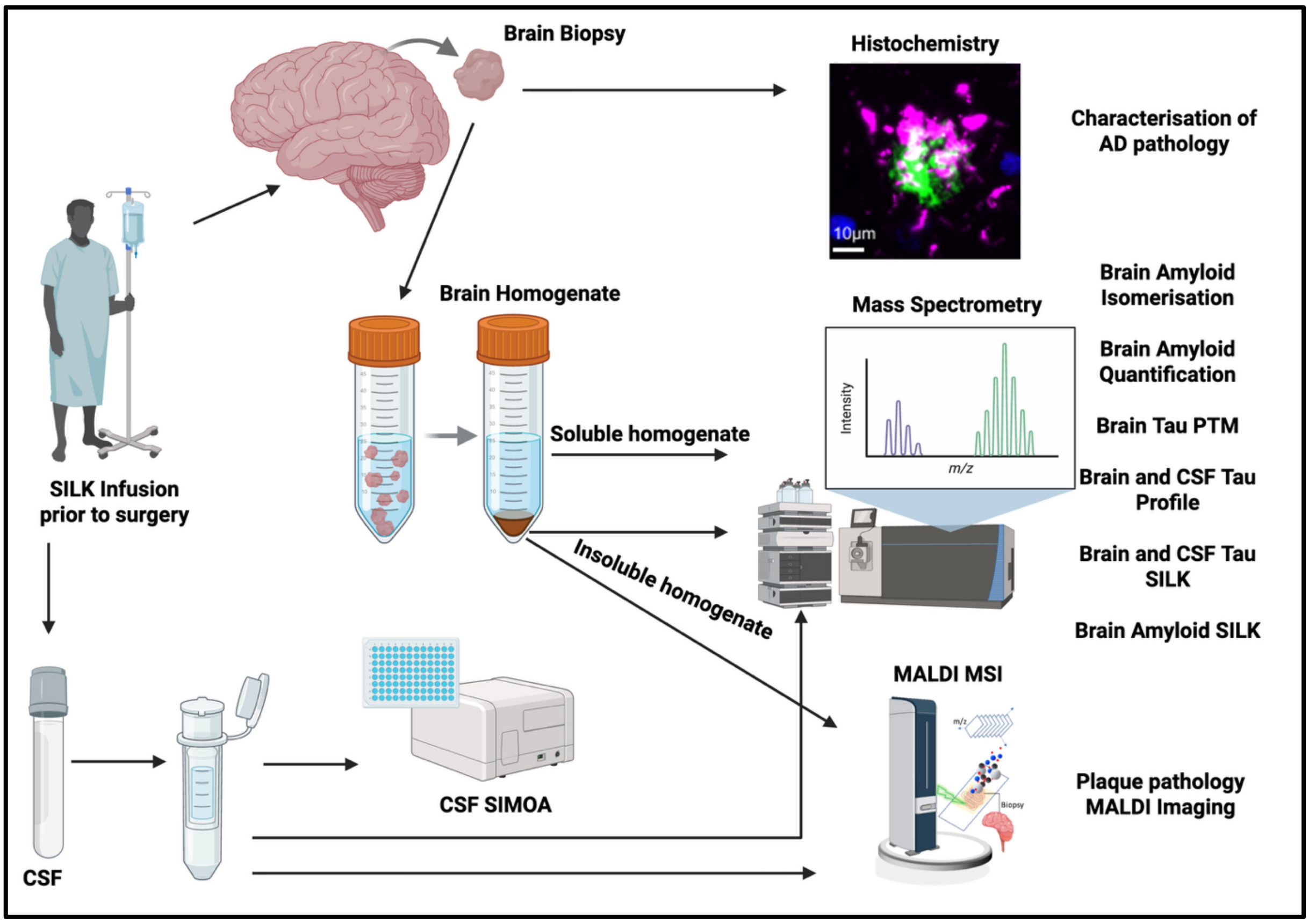

Supplementary Figure 5. The schematic of NPH SILK pipeline and its linked procedures.

Created in BioRender. Aslanyan A (2026) https://BioRender.com/pccznys.

Abbreviations: AD, Alzheimer disease; CSF, Cerebrospinal fluid; MALDI, Matrix-assisted laser desorption/Ionization; MSI, Mass Spectrometry Imaging; PTM, Post-translational modifications; SILK, Stable isotope labelling kinetics; SIMOA, Single molecule array.

Supplementary Tables

Supplementary Table 1. Demographics and neuropathological annotations in the control cohort which includes control participants (Control 1 and 2), participants with AD (AD 1 and 2), CBD and participants with positive amyloid status (Amyloid+ 1 and 2). Adapted from Horie et al, 2020 (DOI: https://doi.org/10.1186/s40478-020-01019-z)

Abbreviations: AD, Alzheimer disease; CBD, Corticobasal Degeneration; PMI, Post-mortem interval between death and freezing of brain tissue; NIA-AA, Histopathological assessment of Aβ deposits (A) and staging of neurofibrillary tangles (B); ApoE, Apolipoprotein E.

	Control 1	Control 2	Amyloid+ 1	Amyloid+ 2	AD 1	AD 2	CBD
Age at death	80	87	90	72	85	81	59
Sex	Male	Male	Male	Male	Male	Female	Male
PMI	7	47	14.5	33.3	16.3	12	48
NIA-AA	A0B1	A0B1	A3B1	A2B1	A3B3	A3B3	N/A
ApoE genotype	ε3/ε3	ε3/ε3	ε3/ε3	ε3/ε4	ε3/ε3	ε3/ε3	N/A

Supplementary Table 2. Clinical presentation, neurological examination findings, pre-shunt brain imaging scores, intravenous leucine dose and clinical follow-up outcomes in individuals with suspected NPH undergoing brain biopsy.

Abbreviations: GCA, Global Cortical Atrophy Scale; iNPH, iNPH Radscale; MTA, Medial Temporal Lobe Atrophy score.

	Presenting complaint	Sex	Cognitive symptoms	Positive neurological examination findings	Pre-shunt Brain Imaging findings	Total IV leucine administered (g)	Clinical follow-up
**Subject 1**	Brain fog<br>Balance problems<br>Urinary and faecal incontinence<br>Falls	M	Executive Episodic<br>Topographical<br>Language	Shuffling gait<br>Cerebellar signs	MTA 2<br>GCA 0<br>Fazekas 0<br>iNPH 8	1.35	Improvement in balance and falls, incontinence and cognition at 3 years
**Subject 2**	Balance problems<br>Falls<br>Word finding difficulty	M	Language	Shuffling gait	MTA 2<br>GCA 0<br>Fazekas 3<br>iNPH 10	0.57	Subjective improvement in balance and cognition which declined after 2 years
**Subject 3**	Shuffling gait<br>Urinary incontinence<br>Falls	F	Executive Episodic<br>Topographical<br>Language	Shuffling gait	MTA 1<br>GCA 1<br>Fazekas 0<br>iNPH 4	1.51	Subjective improvement in cognition and gait in the first 2 months followed by steady decline
**Subject 4**	Balance problems<br>Falls<br>Urinary incontinence	M	Episodic	Shuffling gait	MTA 1<br>GCA 2<br>Fazekas 0<br>iNPH 8	0.95	Improvement in gait and cognition at 1 year followed by decline
**Subject 5**	Balance problems<br>Falls<br>Tinnitus<br>Memory complaints	M	Executive Episodic	Broad based and shuffling gait<br>Pyramidal signs	MTA 3<br>GCA 2<br>Fazekas 1<br>iNPH 9	0.46	Improvement in symptoms, remained stable at last follow-up at 1.5 years.
**Subject 6**	Falls<br>Urinary incontinence	M	Executive<br>Episodic	Shuffling gait with decreased arm swing<br>Parkinsonism<br>Cerebellar signs	MTA 1<br>GCA 0<br>Fazekas 0<br>iNPH 7	0.63	Objective improvement in gait and balance. Stable at 2.5 years.
**Subject 7**	Balance problems<br>Falls<br>Urinary incontinence	F	Episodic	Unsteady gait with short steps	MTA 1<br>GCA 1<br>Fazekas 0<br>iNPH 6	Not labelled	Remarkable improvement in mobility at 6 months.
**Subject 8**	Forgetfulness	M	Episodic	Antalgic gait	MTA 1<br>GCA 0<br>Fazekas 1<br>iNPH 7	Not labelled	No improvement at 8 months.

**Subject 9**	Forgetfulness<br>Falls<br>Urinary incontinence	F	Episodic	Shuffling and slow gait, poor balance	MTA 2<br>GCA 0<br>Fazekas 3<br>iNPH 10	Not labelled	Improvement in gait and balance at 9 months.
**Subject 10**	Balance problems<br>Falls<br>Short term memory complaints<br>Urinary incontinence	F	Episodic<br>Executive	Unable to walk	MTA 2<br>GCA 0<br>Fazekas 2<br>iNPH 7	Not labelled	No changes at 5 months.
**Subject 11**	Short term memory<br>Reasoning skills<br>Gait disturbance<br>Falls<br>Urinary incontinence	M	Executive<br>Episodic<br>Topographical<br>Language	Parkinsonism<br>Hypophonia<br>Supranuclear gaze palsy<br>Shuffling gait	MTA 3<br>GCA 1<br>Fazekas 1<br>iNPH 7	Not labelled	Continued improvement in mobility and cognition at 3 months followed by increased falls at 6 months (still fewer falls than pre-shunt)
**Subject 12**	Short term memory<br>Falls<br>Urinary incontinence	M	Executive<br>Episodic<br>Topographical<br>Visuospatial	Shuffling gait	MTA 2<br>GCA 0<br>Fazekas 1<br>iNPH 10	Not labelled	Improvement in walking at 2 months followed by decline at 8 months
**Subject 13**	Gait disturbance<br>Urinary incontinence	M	Executive<br>Episodic	Unable to walk	MTA 2<br>GCA 0<br>Fazekas 3<br>iNPH 10	Not labelled	No significant improvement in mobility at 6 months
**Subject 14**	Word finding difficulties<br>Misplacing items<br>Gait disturbance<br>Falls<br>Urinary incontinence	F	Episodic	Normal examination	MTA 2<br>GCA 0<br>Fazekas 2<br>iNPH 3	0.66	Subjective improvement in walking at 2 months
**Subject 15**	Gait disturbance<br>Falls<br>Urinary incontinence	M	Episodic	Parkinsonism<br>Apraxia of gait	MTA 2<br>GCA 0<br>Fazekas 3<br>iNPH 8	0.53	Not had follow-up yet
**Subject 16**	Gait disturbance<br>Falls<br>Urinary incontinence	F	Executive	Fragmented vertical saccades<br>Rigidity lower limbs<br>Magnetic left foot	MTA 2<br>GCA 0<br>Fazekas 0<br>iNPH 8	0.44	Improvement in gait, cognition and bladder control at 2 months
**Subject 17**	Forgetfulness<br>Falls<br>Urinary incontinence	M	Executive<br>Episodic<br>Topographical	Shuffling, slow gait	MTA 3<br>GCA 0<br>Fazekas 0<br>iNPH 7	0.55	No significant improvement at 1 month
**Subject 18**	Gait disturbance	M	Executive	Intention tremor<br>Slow gait<br>Mild gait apraxia	MTA 2<br>GCA 0<br>Fazekas 0<br>iNPH 10	0.70	Significant improvement in walking at 3 months

Supplementary Table 3. Data table with normalized ^{13}C leucine labelled peptide compared to endogenous unlabelled peptide for NPH patient brains with measurable quantity of Aβ plaques compared to serial dilution standard curve using a synthetic ^{13}C leucine labelled $Aβ_{1-42}$.

D1-D6 corresponds to Dilution 1-6 used for the IP-MS using similar brain homogenates in a non-labelled AD brain pool.

All "BELOW LOD" labels are detected signal strength that are $>10^2$ below our determined LOD. "NOT DETECTED" labels indicate 0 signal was detected.

Abbreviations: Aβ, β-Amyloid; AD, Alzheimer disease; IP-MS, Immunoprecipitation-Mass Spectrometry; LOD, Limit of detection; LOQ, limit of quantitation; NPH, normal pressure hydrocephalus.

**ID**	**KLVFFAEDVGSN (mid-domain)** $^{13}C/^{12}C$	**KGAIIGLMVGGVV (Aβ1-40)** $^{13}C/^{12}C$	**KGAIIGLMVGGVVIA (Aβ1-42)** $^{13}C/^{12}C$
D1	0.716766974	0.069433194	0.904358657
D2	0.44235462	0.030038142	0.344792474
D3	0.057621088	0.005559101	0.056806595
D4	0.020566893	0.001646362	0.018724334
D5	0.003561521	NOT DETECTED	0.003414295
D6	0.000571668	NOT DETECTED	0.000917069
Subject 3	BELOW LOD	BELOW LOD	BELOW LOD
Subject 8	NOT DETECTED	NOT DETECTED	BELOW LOD
Subject 10	NOT DETECTED	BELOW LOD	BELOW LOD
Subject 11	NOT DETECTED	NOT DETECTED	BELOW LOD
Subject 12	BELOW LOD	NOT DETECTED	BELOW LOD
Subject 15	BELOW LOD	NOT DETECTED	BELOW LOD

Subject 16	BELOW LOD	NOT DETECTED	BELOW LOD

Supplementary Table 4. The estimated LOD/LOQ expressed in percentages for each Aβ peptide for their respective ^{13}C leucine label.

Abbreviations: Aβ, β-Amyloid; LOD, Limit of detection; LOQ, limit of quantitation

	**KLVFFAEDVGSN (mid-domain)** $^{13}C/^{12}C$	**KGAIIGLMVGGVV (Aβ1-40)** $^{13}C/^{12}C$	**KGAIIGLMVGGVVIA (Aβ1-42)** $^{13}C/^{12}C$
**LOD**	2.0566893%	3.0038142%	1.8724334%
**LOQ**	5.7621088%	6.9433194%	5.6806595%

Supplementary Table 5. Overview of sample availability across the NPH and post-mortem cohorts. Schematic summary of which samples were included in each experimental component of the article. Tick marks indicate assays/analysis performed for each subject across ex vivo brain, CSF and clinical data.

	Brain ex vivo								CSF				Clinical details
	Histo-chemistry	MALDI	Amyloid isomerisation	Amyloid quantification	Tau PTM	Tau Profile	Tau SILK	Amyloid SILK	Ventricular CSF SIMOA	Ventricular CSF Tau Profile	Lumbar CSF Tau Profile	Tau SILK	
**NPH Cohort**													
Subject 1	☑	☑	☑	☑	☑	☑	☑	☐	☑	☐	☑	☑	☑
Subject 2	☑	☑	☑	☑	☑	☑	☑	☐	☑	☐	☑	☑	☑
Subject 3	☑	☑	☑	☑	☑	☑	☑	☑	☑	☑	☑	☑	☑
Subject 4	☑	☑	☑	☑	☑	☑	☑	☐	☑	☑	☑	☑	☑
Subject 5	☑	☑	☑	☑	☑	☑	☑	☐	☑	☑	☑	☑	☑
Subject 6	☑	☑	☑	☑	☑	☑	☑	☐	☑	☑	☑	☑	☑
Subject 7	☑	☑	☐	☐	☐	☐	☐	☐	☑	☑	☑	☐	☑
Subject 8	☑	☑	☑	☑	☑	☐	☐	☑	☑	☐	☐	☐	☑
Subject 9	☑	☑	☑	☑	☑	☐	☐	☐	☑	☐	☐	☐	☑
Subject 10	☑	☑	☑	☑	☑	☐	☐	☑	☑	☐	☐	☐	☑
Subject 11	☑	☑	☑	☑	☑	☐	☐	☑	☑	☐	☐	☐	☑
Subject 12	☑	☑	☑	☑	☑	☐	☐	☑	☑	☐	☐	☐	☑
Subject 13	☑	☑	☑	☑	☑	☐	☐	☐	☑	☐	☐	☐	☑
Subject 14	☑	☐	☑	☑	☑	☐	☑	☐	☑	☐	☐	☐	☑
Subject 15	☑	☐	☑	☑	☑	☐	☑	☑	☑	☐	☐	☐	☑
Subject 16	☑	☐	☑	☑	☑	☐	☑	☑	☑	☐	☐	☐	☑
Subject 17	☑	☐	☑	☑	☑	☐	☑	☐	☑	☐	☐	☐	☑
Subject 18	☑	☐	☑	☑	☑	☐	☑	☐	☑	☐	☐	☐	☑
**Post-mortem Cohort**													
Control 1	☐	☐	☑	☑	☑	☑	☑	☐	☐	☐	☐	☐	☑
Control 2	☐	☐	☑	☑	☑	☑	☑	☐	☐	☐	☐	☐	☑
Amyloid+ 1	☐	☐	☑	☑	☑	☑	☑	☐	☐	☐	☐	☐	☑
Amyloid+ 2	☐	☐	☑	☑	☑	☑	☑	☐	☐	☐	☐	☐	☑
AD 1	☐	☐	☑	☑	☑	☑	☑	☐	☐	☐	☐	☐	☑
AD 2	☐	☐	☑	☑	☑	☑	☑	☐	☐	☐	☐	☐	☑
CBD	☐	☐	☑	☑	☑	☑	☑	☐	☐	☐	☐	☐	☑
