# Supplementary material for: Spatiotemporal characterization of Alzheimer disease pathology in living human brain tissue
Source: Nat Commun. 2026 Sep 24;17:9878. doi: 10.1038/s41467-026-76494-4 (PMC13612511; doi:10.1038/s41467-026-76494-4)
Supplement: Supplementary file 2 [file 41467_2026_76494_MOESM2_ESM.pdf]

nature portfolio

Peer Review File

Spatiotemporal characterization of Alzheimer disease pathology in living human brain tissue

Corresponding Author: Dr Aram Aslanyan

This file contains all reviewer reports in order by version, followed by all author rebuttals in order by version.

Version 0:

Reviewer comments:

Reviewer #1

(Remarks to the Author)
Aslanyan et al. report an extensive analysis of 6 biopsy cases for AD pathology from NPH patients receiving shunt surgery. The patients received 13C leucine infusions prior to surgery to determine the turnover of tau and Abeta. The isotopes were identified by mass spectrometry. In addition, mass spectrometry imaging was used to determine the composition of amyloid plaques. The authors looked for Abeta peptides of different length, with post-translational modifications, such as 3pGlu and isomerization. The mass spectrometry imaging was supplemented by histochemistry (LCO) and immunohistochemistry. The results from the biopsy samples were supplemented with 7 autopsy cases. The main findings follow two different and independent directions: 1. Tau turnover and composition, and 2. Abeta plaque composition and maturation.
1. Regarding the tau turnover and composition, the authors found that tau has a half-life of 12 days in the brain and that brain tau is closer to ventricular CSF than lumbar CSF.
2. With respect to Abeta plaques the main finding is an increase of isomerized Abeta forms and 3pGlu Abeta in two AD cases compared to the NPH cases. Here, only in 4 out 6 amyloid pathology was found. Tau pathology was not observed microscopically in the NPH biopsies.
The methodological approaches are excellent and appear to be well performed. However, the number of cases is very low. Other groups working with NPH biopsies usually publish larger cohorts. To address the shortness of cases, 7 additional autopsy brains were included. However, for the question of plaque maturation even this number appears to me not sufficient. Moreover, the combination of two scientific studies in one manuscript makes it difficult to figure out whether there is more than just descriptive results. The authors may consider giving this study a better visible focus.
The results about the tau and Abeta turnover and the comparison of tau profiles from brain tissue with ventricular and lumbar CSF are novel, important, and relevant for the interpretation of the results that can be obtained from lumbar punctures.
The result about the plaque composition and maturation with an increase isomerized Abeta in more mature plaques is also novel but just adds another piece to the sequence of biochemical abeta plaque maturation (PMID: 9173910; 24519982). A more general picture of plaque maturation should be elaborated in the discussion including the relevant literature about Abeta aggregate maturation.
Specific points:
- The number of cases is very low. This is a major limitation of this study! An increased n would help, probably by adding more autopsy cases.
- Tau and Abeta turnover: This experiment was carried out in NPH patients without visible tau pathology as indicated by a NFT density of 0 NFT/mm². How relevant can these findings be for NFT tau?
- Abeta in plaques showed no major 13C leucine uptake and were considered as stable structures. Data about the 13C leucine enrichment of Abeta were described as “data not shown”. The results should be documented, at least in the supplementary material.
- Regarding the plaque maturation and the pattern of 3pGlu Abeta the positivity in the plaque halo was also seen in previously published images (PMID: 9173910, 7857653, 8952519 and others) as well as in the soluble fraction of brain lysates from AD patients (PMID: 24519982). This limits the novelty of the descriptive 3pGlu Abeta finding.
- Interactions between tau and Abeta have been shown earlier. In a functional manner in animals that showed increased tau pathology after increase of Abeta (PMID: 11520988, 11520987) as well as by immunoprecipitation and proximity ligation assays (PMID: 31414210). To what extent do the presented findings extent these previous findings? Confirmation of these earlier studies in biopsies would be of limited novelty.
- The authors claim that they report AD changes “in vivo”. This is not true. They analyzed biopsies which represents tissue that has been removed from “in vivo”/ living patients by surgery and was studied “ex vivo” with biochemical and histological

methods.
In my opinion the term “in vivo” should be reserved to experiments in living individuals. An example for an “in vivo” experiment would be the analysis of in vivo pathology with two photon microscopy through a cranial window on multiple days while the animal lives and can be exposed to different conditions.
- What was the clinical trial identifier for the application of 13C leucine to NPH patients prior to surgery? Please mention this in the methods section. As far as I know 13C leucine infusions do not represent a clinical standard procedure prior to NPH shunt surgery.

Reviewer #2

(Remarks to the Author)
This is a unique study combining SILK with brain biopsy in people undergoing shunt surgery for NPH. The major limitation is (as the authors acknowledge) the small sample size (with only one case with high density of Aβ plaques), which potentially question the generalizability of some of the findings.

To me, one of the main findings are that amyloid-beta plaques does not seem to be very dynamic structures. Does this finding challenge the hypothesis that low levels of Aβ42 in CSF is just a consequence of most Aβ42 getting stuck in the plaques?

Another main finding was that the half-life of tau in brain tissue is about 12 days. How ever, the data points used to establish the model are very few (Figure 3). How uncertain is the estimation of the hafl-life of tau? Do the authors now have access to more cases that could be used in this modelling approach?

Another very important finding was that the production rate of tau might increase with the number of amyloid-beta plaques, but again the data points are too scarce to make any firm conclusion. Do the authors now have access to more cases?

The authors might consider citing previous papers that have shown that CSF AD biomarkers do not detect the very earliest stages of AD when amyloid-plaques are just emerging (e.g. PMID: 12601108; PMID: 35173015).

In general, the layout of the figures and the figure legends can be improved (e.g describing all abbreviations used in the figures). The discussion could also be more focused.

In summary, the paper is very novel and provide important new insights, but if no more cases can be added, the author should even more stress that some findings are very preliminary and need to be confirmed in larger studies in the future.

Reviewer #3

(Remarks to the Author)
In this study, Aslanyan et al. use a novel SILK isotope labelling methodology to examine the kinetics of tau protein in brain and CSF, and also examine the relative turnover of beta-amyloid plaques. Additional MS methodology is used to better characterize the components of tau and amyloid fragments in AD brain. Patients with NPH and co-morbid AD pathology are used to ensure that the SILK data is analyzed at a timepoint that gives more information than autopsy tissue, where the isotope labeling happens at a distant time point from tissue acquisition. While this study has many novel aspects, there are some significant weaknesses. As the authors point out in the Discussion, the low sample number of NPH patients (6 total) greatly limits this study. This may be why the figures are largely descriptive. Statistical analysis to back up the claims in this paper should be made wherever possible.

Specific examples:

Figure 1: “pTau217-positive neurites 536 are found to be associated with amyloid plaques” – is there any way to quantify overlap?

Figure 2: “C-terminus for NPH samples 543 show trend towards higher abundance compared to post-mortem samples” – is this trend significant?

Figure 4: G through N purports to show different fragments localized to different areas of plaques, but the images all look very similar. Would it be possible to show an overlay of some of these images to better make the case that these fragments are in different plaque areas?

The first sections states: “Early brain amyloid deposition occurs before CSF biomarker conversion.” This statement is based on the fact that 2 of their patients with beta-amyloid pathology did not have CSF biomarker conversion. But it is difficult to extrapolate from this data, given that we have no idea how common this is in a larger population, or how much beta-amyloid is even in the CNS of these “divergent” patients (did the biopsy happen to hit an area of brain with early beta-amyloid pathology?). The fact that this happened in 2 of the 4 patients with beta-amyloid pathology is suggestive, but with an effective n of 4 total its hard to know how representative this is. It would also be interesting to check the ventricular CSF for biomarker values, which the authors themselves say is more representative of brain pathophysiology.

Minor Points:

Abstract – there needs to be some kind of summary at the end that helps the reader understand the overall importance of these findings.

Lines 179 – 185 – what exactly do the divergent ratios of 4R/3R imply? It seems that less new 4R is made vs. 3R, but overall there is more 4R than 3R.

Lines 258 – 260 – it sounds like you are claiming that beta-amyloid 40 constitutes the halo of plaques, but your own data states otherwise (lines 229-231) as does the literature.

Reviewer #4

(Remarks to the Author)
The study Aslanyan et al. provides new insights into the early stages of Alzheimer´s disease (AD) and highlights the importance of sensitive biomarkers and innovative methodologies for advancing diagnosis and treatment. The study's approach of using in vivo brain tissue and comparing it with post-mortem samples offers a comprehensive view of the progression of AD. Their results showed that brain amyloid pathology can be captured very early, even before lumbar CSF biomarkers become positive. A small number of plaques were found in individuals without positive lumbar CSF biomarker profiles, suggesting early microscopic accumulation of Aβ. Also the findings on tau kinetics and amyloid plaque composition provide valuable insights for future AD research and clinical trials. They used large number of sophisticated methods that are sound and well described. Overall the manuscript is well written and could be considered for publication in NC.

I have few comments and suggestions:
The small sample size and specific clinical status (NPH patients) limit the generalizability of the findings. Further studies are needed to confirm these findings in a larger population. The discussion and conclusions should be modified in this way.

#The conclusions about the half life of tau in the brain tissue was based on the investigation of the single area of the brain and does not necessary apply to all brain areas.

The labeling uptake in ventricular CSF was observed within a short time frame of under 5 hours. This limited observation period may not capture the complete dynamics of tau secretion and distribution, potentially overlooking longer-term patterns and variations.

Given the correlation between tau labeling and amyloid plaques was not statistically significant, consider expanding the study cohort or employing advanced statistical techniques to strengthen the analysis.

The low tracer uptake in lumbar CSF suggests a need for higher doses or more sensitive detection methods to better capture tau dynamics in this compartment.

The difference in the 4R:3R ratio across different cohorts suggests that tau isoform distribution might influence labeling patterns and could be explored further to understand its implications for tauopathies.

Tracking the subject throughout the manuscript is quite hard. It might be helpful if the authors could include a schematic picture in the supplementary material that outlines which experiments were conducted on which subjects.

In Fig 1 please describe in caption what was the method used for the pT217 staining

Version 1:

Reviewer comments:

Reviewer #1

(Remarks to the Author)
This is a previously submitted manuscript that had been revised. Most importantly, the authors were able to increase their sample size significantly. The results that are published appear reliable and are properly discussed.
Minor points:
- Please adjust the format of Tab. 1 and Suppl. Fig. 5. Please organize the supplementary material in a reader friendly manner (1 figure or table on one page)!
- Please indicate in the methods section that 13CLeucine is considered as a non investigational medicinal product by

MHRA meaning that giving the product to patients is not considered as a clinical trial. Just for transparency.

Reviewer #3

(Remarks to the Author)
This manuscript is greatly improved from the prior submission, and all of my original comments have been addressed. On the whole, this is a significant contribution to the field and I think worthy of publication. However, given the major revisions that have gone into this work, I have several new concerns that need to be addressed.

1) It appears that the half-life of brain tau that was in the original publication (12 days) has been significantly adjusted (to 34 days). In the response to another reviewer's comments, the authors explain that this is due to an updated plasma leucine model that now incorporates more patient data. This is completely understandable and admirable that they are attempting to be as accurate as possible. However, this highlights how important the details of this model are to this major conclusion of the paper. Perhaps I missed it, but I was unable to find a detailed description of the model and the assumptions that went into it in their methods. Given how important this is, this should be described in detail.

2) In Figure 3B – the ventricular CSF group has a very wide spread, and only three datapoints; wouldn't it make sense to include more of your samples to make this more significant? I'm not sure how many of the 18 NPH patients in this study have ventricular CSF you can use, but if possible this would help buttress this finding.

3) In Figure 4A – it seems odd to draw a curve based on two data points significantly beyond 0 (why didn't you do this for 4B?). To me these isolated points don't suggest a curve, but simply that the levels stay elevated for some time afterwords, with a suggestion of a later decline.

4) Lines 440-443 – A minor point, but I think contrasting the mechanism of beta-amyloid accumulation in NPH with all other non-NPH AD cases is an unnecessarily dichotomy. Beta-amyloid accumulation in AD has been proposed to be related to a range of processes including infection, brain trauma, ApoE4 physiology, and vascular dysfunction to give some examples (and then of course there is familial AD). I would simply say that altered CSF dynamics is likely one of many ways to contribute to beta-amyloid accumulation.

Version 2:

Reviewer comments:

Reviewer #3

(Remarks to the Author)
All of my concerns have been addressed - I commend the authors on an excellent study.

Open Access This Peer Review File is licensed under a Creative Commons Attribution 4.0 International License, which

permits use, sharing, adaptation, distribution and reproduction in any medium or format, as long as you give appropriate credit to the original author(s) and the source, provide a link to the Creative Commons license, and indicate if changes were made.
In cases where reviewers are anonymous, credit should be given to 'Anonymous Referee' and the source.
The images or other third party material in this Peer Review File are included in the article's Creative Commons license, unless indicated otherwise in a credit line to the material. If material is not included in the article's Creative Commons license and your intended use is not permitted by statutory regulation or exceeds the permitted use, you will need to obtain permission directly from the copyright holder.
To view a copy of this license, visit https://creativecommons.org/licenses/by/4.0/

RESPONSE TO REVIEWERS' COMMENTS

Aslanyan et al. report an extensive analysis of 6 biopsy cases for AD pathology from NPH patients receiving shunt surgery. The patients received 13C leucine infusions prior to surgery to determine the turnover of tau and Abeta. The isotopes were identified by mass spectrometry. In addition, mass spectrometry imaging was used to determine the composition of amyloid plaques. The authors looked for Abeta peptides of different length, with post-translational modifications, such as 3pGlu and isomerization. The mass spectrometry imaging was supplemented by histochemistry (LCO) and immunohistochemistry. The results from the biopsy samples were supplemented with 7 autopsy cases. The main findings follow two different and independent directions: 1. Tau turnover and composition, and 2. Abeta plaque composition and maturation.

1. Regarding the tau turnover and composition, the authors found that tau has a half-life of 12 days in the brain and that brain tau is closer to ventricular CSF than lumbar CSF.

2. With respect to Abeta plaques the main finding is an increase of isomerized Abeta forms and 3pGlu Abeta in two AD cases compared to the NPH cases. Here, only in 4 out 6 amyloid pathology was found. Tau pathology was not observed microscopically in the NPH biopsies. The methodological approaches are excellent and appear to be well performed. However, the number of cases is very low. Other groups working with NPH biopsies usually publish larger cohorts. To address the shortness of cases, 7 additional autopsy brains were included. However, for the question of plaque maturation even this number appears to me not sufficient. Moreover, the combination of two scientific studies in one manuscript makes it difficult to figure out whether there is more than just descriptive results. The authors may consider giving this study a better visible focus.

We thank the reviewer for their valuable comments. We have now increased the number of cases substantially, now including a total of 18 ex vivo biopsies and 7 post-mortem brain samples. We have now also added a schematic summary (SM Table 5) and schematic of NPH pipeline (SM Figure 5) which should help guide the readers through the scientific studies that were done on each sample.

The results about the tau and Abeta turnover and the comparison of tau profiles from brain tissue with ventricular and lumbar CSF are novel, important, and relevant for the interpretation of the results that can be obtained from lumbar punctures.

The result about the plaque composition and maturation with an increase isomerized Abeta in more mature plaques is also novel but just adds another piece to the sequence of biochemical abeta plaque maturation (PMID: 9173910; 24519982). A more general picture of plaque maturation should be elaborated in the discussion including the relevant literature about Abeta aggregate maturation.

We have now added this in our revised manuscript.

Amyloid burden does not always correlate with disease severity or cognitive impairment in sporadic or familial AD (70, 71). Biochemical staging of amyloid plaques is based on the heterogeneity of Aβ isoforms and modified species that could be detected with AD patients compared to pathologically preclinical AD cases (72). At the earliest stages $A\beta_{42}$ aggregates to form diverse form of plaques, with time other Aβ isoforms aggregates and accumulation of multiple PTMs on Aβ takes place (73, 74). Plaque age, and the structural maturity are crucial factors how an initial nidus could impart neurotoxicity with time (45, 75).

Specific points:

- The number of cases is very low. This is a major limitation of this study! An increased n

would help, probably by adding more autopsy cases.

We have now added 12 more NPH brain samples bringing the total number to 18 NPH ex vivo brain samples (11/18 SILK labelled) and 7 post-mortem brain samples. We believe this significantly improves this work and the value of the conclusions.

- Tau and Abeta turnover: This experiment was carried out in NPH patients without visible tau pathology as indicated by a NFT density of 0 NFT/mm². How relevant can these findings be for NFT tau?

We have now added a column showing the tissue area positive for pTau217 staining (%) in Table 1. Since our samples are likely reflective of early AD pathology most of our samples do not have evidence of NFT, however pTau217 staining (Table 1 and Figure 1) shows that there is already an ongoing tau pathology.

- Abeta in plaques showed no major 13C leucine uptake and were considered as stable structures. Data about the 13C leucine enrichment of Abeta were described as "data not shown". The results should be documented, at least in the supplementary material.

We have now added SM Tables 3 and 4 which show serial dilution results and estimated limits of detection and quantitation.

- Regarding the plaque maturation and the pattern of 3pGlu Abeta the positivity in the plaque halo was also seen in previously published images (PMID: 9173910, 7857653, 8952519 and others) as well as in the soluble fraction of brain lysates from AD patients (PMID: 24519982). This limits the novelty of the descriptive 3pGlu Abeta finding.

All cited papers have autopsy cases, our study is unique as we use ex vivo brain tissue of living participants. Furthermore, this is the first description of the mass spectrometry-based technique (MALDI) for detailed characterisation that is not dependent on specific antibodies, therefore an important validation.

- Interactions between tau and Abeta have been shown earlier. In a functional manner in animals that showed increased tau pathology after increase of Abeta (PMID: 11520988, 11520987) as well as by immunoprecipitation and proximity ligation assays (PMID: 31414210). To what extent do the presented findings extent these previous findings? Confirmation of these earlier studies in biopsies would be of limited novelty.

We thank the reviewer for highlighting these previous studies, and that referencing these earlier works gives our work more context. The reviewer correctly points out that interactions between tau and Abeta in animal models (PMID: 11520988, 11520987) and autopsy (PMID: 31414210) cases has previously been observed. Our study shows this for the first time in *ex-vivo* human brain of living individuals. It is extremely important that the field can validate the important findings that this reviewer references, but being able to show this in living people, at the relevant stage of the disease provides the ground truth of what is actually happening in the brain.

- The authors claim that they report AD changes "in vivo". This is not true. They analyzed biopsies which represents tissue that has been removed from "in vivo"/ living patients by

surgery and was studied “ex vivo” with biochemical and histological methods.
In my opinion the term “in vivo” should be reserved to experiments in living individuals. An example for an “in vivo” experiment would be the analysis of in vivo pathology with two photon microscopy through a cranial window on multiple days while the animal lives and can be exposed to different conditions.

We agree with reviewer’s comment – “in vivo” has been changed to “ex vivo” in the manuscript.

- What was the clinical trial identifier for the application of 13C leucine to NPH patients prior to surgery? Please mention this in the methods section. As far as I know 13C leucine infusions do not represent a clinical standard procedure prior to NPH shunt surgery.

Clinical trial identifier is not required as 13C Leucine is considered non investigational medicinal product (NIMP) by the MHRA. However, the study was approved by the Bloomsbury ethics committee and all individuals provided informed written consent as mentioned in the methods (Experimental Model and Study Participant Details). We can also provide written confirmation of the NIMP status of the stable isotope from the MHRA if the editor believes this is necessary.

Reviewer #2 (Remarks to the Author):

This is a unique study combining SILK with brain biopsy in people undergoing shunt surgery for NPH. The major limitation is (as the authors acknowledge) the small sample size (with only one case with high density of Aβ plaques), which potentially question the generalizability of some of the findings. To me, one of the main findings are that amyloid-beta plaques does not seem to be very dynamic structures. Does this finding challenge the hypothesis that low levels of Aβ42 in CSF is just a consequence of most Aβ42 getting stuck in the plaques?

We thank the reviewer for their valuable comments. Our findings do not contradict the hypothesis of low levels of Aβ42 in CSF being caused by it getting stuck in plaques, but we are adding new information to this story by providing new information about the tempo of this process. Our data suggests that the turnover is very slow (since it’s likely forming over years); our method is sensitive with very low limits of detection for labelled Aβ42, but not sensitive *enough* to capture the slow turnover. It is important to remind the reader that with SILK we are only observing a snapshot of a few hours of Aβ42 translation/ potential plaque incorporation in plaques that may have been evolving over many months / years. This is consistent with the Aβ isomerization data which also suggests plaques are established.

Another main finding was that the half-life of tau in brain tissue is about 12 days. However, the data points used to establish the model are very few (Figure 3). How uncertain is the estimation of the half-life of tau? Do the authors now have access to more cases that could be used in this modelling approach?

We have taken this reviewer’s point very seriously and we have now labelled additional cases over the past year to be certain of our findings. We are pleased to report that we now have 5 additional SILK labelled samples (total of 11 SILK labelled samples) which are added to the data set to establish the half-life model (see Figure 4D). We acknowledge that we are not able

to capture the full tau SILK curve because it was not clinically feasible to collect brain biopsies at all relevant timepoints after SILK labelling, and for ethical reasons we had to work around the participant's scheduled clinical neurosurgical care.

Since the original submission in summer 2024, we have updated the pharmacokinetic model used to estimate tau turnover (TTR half-life) in brain. The original approach relied on a plasma leucine kinetics curve derived from a single individual, which disproportionately influenced the tau half-life. We have now replaced this with an updated plasma leucine model using data from more than 100 participants that were not available in 2024, improving the stability and generalisability of the model. Using this revised model, the estimated brain tau half-life is 33.6 days. We recognise this represents a significant change from our prior estimate (approximately 12 days), however we consider the revised estimate more credible because it is informed by a much larger evidence base for plasma leucine kinetics and yields turnover estimates that are more consistent with published CSF tau kinetics (e.g. Sato et al., 2018). The manuscript describing the pharmacokinetic model is in preparation and the tau half-life that is reported here is consistent with what will be reported in that manuscript.

We acknowledge in the discussion that these results are preliminary in brain tissue, and more data at relevant timepoints would be needed to confirm our findings.

Another very important finding was that the production rate of tau might increase with the number of amyloid-beta plaques, but again the data points are too scarce to make any firm conclusion. Do the authors now have access to more cases?

Again, we were very encouraged by the reviewer's interest in this point and set about trying to address this question with new cases, collected prospectively. With the additional SILK labelled samples (increased from n=6 to n=11) we explored correlations between labelled tau peptides and amyloid plaques. We found that the tau peptide 212-221 TTR strongly correlated with plaques (r=0.76). Although the correlation was not statistically significant once corrected for multiple comparisons (Spearman's p; Benjamini-Hochberg across n=5 peptide groups; p=0.053), this is likely due to limited number of available samples and the stringency of our correction methods. We believe that we report this in a balanced way in the manuscript, and that this finding should now be validated at scale.

The authors might consider citing previous papers that have shown that CSF AD biomarkers do not detect the very earliest stages of AD when amyloid-plaques are just emerging (e.g. PMID: 12601108; PMID: 35173015).

We thank the reviewer for the suggestion – the papers have now been cited in the introduction.

In general, the layout of the figures and the figure legends can be improved (e.g describing all abbreviations used in the figures). The discussion could also be more focused.

We thank the reviewer for the suggestion – the figures and legends have been changed to a more uniformed style and we have made the discussion more focused.

In summary, the paper is very novel and provide important new insights, but if no more cases can be added, the author should even more stress that some findings are very preliminary and need to be confirmed in larger studies in the future.

We thank the reviewer for the helpful feedback. We have now added 12 additional ex vivo brain samples (including 5 SILK labelled samples) bringing the total to 18 *ex vivo* brain samples (11/18 SILK labelled) and believe that the manuscript has been greatly strengthened following this advice. We still acknowledge in the discussion that the sample size remains relatively small and that further larger studies should be carried out, however this now represent one of the largest and most comprehensive cohorts of living brain tissue containing AD pathology. It provides an important message to the field that these ambitious clinical protocols involving SILK, sampling of multiple physiological compartments including brain are feasible and have enormous potential, especially when they leverage novel analytical techniques like MALDI.

Reviewer #3 (Remarks to the Author):

In this study, Aslanyan et al. use a novel SILK isotope labelling methodology to examine the kinetics of tau protein in brain and CSF, and also examine the relative turnover of beta-amyloid plaques. Additional MS methodology is used to better characterize the components of tau and amyloid fragments in AD brain. Patients with NPH and co-morbid AD pathology are used to ensure that the SILK data is analyzed at a timepoint that gives more information than autopsy tissue, where the isotope labeling happens at a distant time point from tissue acquisition. While this study has many novel aspects, there are some significant weaknesses. As the authors point out in the Discussion, the low sample number of NPH patients (6 total) greatly limits this study. This may be why the figures are largely descriptive. Statistical analysis to back up the claims in this paper should be made wherever possible.

We thank the reviewer for acknowledging the novelty of this work, and like reviewers 1 and 2 they express concerns about sample size. We hope that we have now addressed these concerns by increasing the number of cases to 18. We have been able to carry out statistical analyses as outlined below given the increased sample size.

Specific examples:

Figure 1: "pTau217-positive neurites 536 are found to be associated with amyloid plaques" – is there any way to quantify overlap?

We were able to look at pTau217 stain enrichment around the neuritic plaque which progressively decreased with distance (Figure 1C). The difference was statistically significant using Kruskal-Wallis test.

Figure 2: "C-terminus for NPH samples 543 show trend towards higher abundance compared to post-mortem samples" – is this trend significant?

We have now shown that the peptide groups in the N-terminus (specifically, peptide groups 25-44 and 45-67) and C-terminus (specifically peptide groups 260-267, 275-280, 354-369) show statistically significant difference between ex vivo NPH and post-mortem brain samples using Mann-Whitney U with Benjamini-Hochberg correction for multiple comparisons.

Figure 4: G through N purports to show different fragments localized to different areas of plaques, but the images all look very similar. Would it be possible to show an overlay of some of these images to better make the case that these fragments are in different plaque areas?

We have adjusted the images of Figure 4 (currently Figure 5) to make them clearer. (G-H) and (J-K) pairs show images of the same area for different Aβ species. (I) and (L) show the overlay of (G-H) and (J-K) respectively, and the pictures on the right show magnified insets of Aβ species in (I) and (L).

The first sections states: "Early brain amyloid deposition occurs before CSF biomarker conversion." This statement is based on the fact that 2 of their patients with beta-amyloid pathology did not have CSF biomarker conversion. But it is difficult to extrapolate from this data, given that we have no idea how common this is in a larger population, or how much beta-amyloid is even in the CNS of these "divergent" patients (did the biopsy happen to hit an area of brain with early beta-amyloid pathology?). The fact that this happened in 2 of the 4 patients with beta-amyloid pathology is suggestive, but with an effective n of 4 total its hard to know how representative this is. It would also be interesting to check the ventricular CSF for biomarker values, which the authors themselves say is more representative of brain pathophysiology.

We have now added the ventricular CSF biomarker values for all our participants instead. The CSF Aβ1-42/40 ratio had good predictive value for detecting amyloid pathology, however the rest of the pilot ventricular biomarkers showed no correlation with plaques. We think this may be due to intraoperative ventricular CSF contamination from the brain tissue and further studies would be needed to explore this.

Minor Points:

Abstract – there needs to be some kind of summary at the end that helps the reader understand the overall importance of these findings.

The abstract has been changed with the summary to show the overall importance of the findings.

Lines 179 – 185 – what exactly do the divergent ratios of 4R/3R imply? It seems that less new 4R is made vs. 3R, but overall there is more 4R than 3R.

Our data suggest that it is possible to quantitate the appearance of peptides that represent 3R and 4R tau in the soluble fraction of human brain. As a proof of concept, we believe this is important, and may be leveraged in future to understand how these isoforms are processed/cleared/incorporated into pathology in different disease states. Given the small sample sizes in this study, we have been cautious about over interpreting these findings. We acknowledge that lower labelling of 4R tau is seen compared to other isoforms, but this may

be due to a number of factors, including synthesis time, rate of incorporation into pathology and so we have discussed this cautiously.

Lines 258 – 260 – it sounds like you are claiming that beta-amyloid 40 constitutes the halo of plaques, but your own data states otherwise (lines 229-231) as does the literature.

We thank the reviewer for pointing this out. The wording has now been changed in agreement with our data and literature.

Reviewer #4 (Remarks to the Author):

The study Aslanyan et al. provides new insights into the early stages of Alzheimer´s disease (AD) and highlights the importance of sensitive biomarkers and innovative methodologies for advancing diagnosis and treatment. The study's approach of using in vivo brain tissue and comparing it with post-mortem samples offers a comprehensive view of the progression of AD. Their results showed that brain amyloid pathology can be captured very early, even before lumbar CSF biomarkers become positive. A small number of plaques were found in individuals without positive lumbar CSF biomarker profiles, suggesting early microscopic accumulation of Aβ. Also the findings on tau kinetics and amyloid plaque composition provide valuable insights for future AD research and clinical trials. They used large number of sophisticated methods that are sound and well described. Overall the manuscript is well written and could be considered for publication in NC.
I have few comments and suggestions:
The small sample size and specific clinical status (NPH patients) limit the generalizability of the findings. Further studies are needed to confirm these findings in a larger population. The discussion and conclusions should be modified in this way.

We thank the reviewer for their comments. We agree and changed the discussion and conclusion to reflect their suggestion.

The conclusions about the half life of tau in the brain tissue was based on the investigation of the single area of the brain and does not necessary apply to all brain areas.

We agree with this observation and this was added in the limitation in discussion and conclusion.

The labeling uptake in ventricular CSF was observed within a short time frame of under 5 hours. This limited observation period may not capture the complete dynamics of tau secretion and distribution, potentially overlooking longer-term patterns and variations.

We agree that our ventricular CSF does not capture longer-term dynamics. We have clarified that ventricular CSF was collected intraoperatively and thus the early detection of tau may have been influenced by procedure related release of brain proteins rather than steady-state secretion and distribution. We now state that confirmation with ventricular CSF collected from non-operative conditions is required to confirm the ventricular CSF characteristics.

Given the correlation between tau labeling and amyloid plaques was not statistically significant, consider expanding the study cohort or employing advanced statistical techniques

to strengthen the analysis.

We collected 5 additional SILK labelled brain samples (total of 11 SILK labelled brain samples). We looked at each available tau peptide group, which showed positive correlation between tau peptide 212-221 and amyloid plaques (r=0.76), however the correlation was not statistically significant once corrected for multiple comparisons (Spearman's ρ; Benjamini-Hochberg across n=5 peptide groups; p=0.053). This is likely due to limited number of available samples.

The low tracer uptake in lumbar CSF suggests a need for higher doses or more sensitive detection methods to better capture tau dynamics in this compartment.

We mentioned in the discussion that higher dose and longer infusion duration (4mg/kg/hr over 16 hours) is required to achieve sufficient labelling of tau in lumbar CSF. As our primary aim was to look at tau production and amyloid plaques in the brain we did not follow the established method for lumbar CSF labelling and gave a smaller dose (2mg/kg/hr) for much shorter duration (up to 9 hours but most of our participants received much shorter infusion instead dictated by clinical care).

The difference in the 4R:3R ratio across different cohorts suggests that tau isoform distribution might influence labeling patterns and could be explored further to understand its implications for tauopathies.

Thank you for the suggestion – we incorporated this in the discussion. Given the very small sample size we cannot draw any meaningful conclusions, but as a proof of concept, we are able to show that it is possible to quantitate the appearance of these isoforms in brain tissue.

Tracking the subject throughout the manuscript is quite hard. It might be helpful if the authors could include a schematic picture in the supplementary material that outlines which experiments were conducted on which subjects.

We have now created a table (SM Table 5) which shows which samples were included for each experiment.

In Fig 1 please describe in caption what was the method used for the pT217 staining

We have now made our figure captions more detailed including Figure 1. It currently reads: "*Representative immunofluorescence images showing the range of AD-type pathology observed across biopsy samples. Amyloid deposits were visualized using LCO staining (green), phosphorylated tau (pTau217) was detected by immunofluorescence (magenta), and nuclei were counterstained with DAPI (blue).*"

RESPONSE TO REVIEWERS' COMMENTS

Reviewer #1 (Remarks to the Author):

This is a previously submitted manuscript that had been revised. Most importantly, the authors were able to increase their sample size significantly. The results that are published appear reliable and are properly discussed.

We thank the reviewer for their comments, and for re-reviewing our manuscript.

Minor points:
- Please adjust the format of Tab. 1 and Suppl. Fig. 5. Please organize the supplementary material in a reader friendly manner (1 figure or table on one page)!

All tables and figures in the supplementary material have been adjusted so there is one figure/table per page.

- Please indicate in the methods section that 13CLeucine is considered as a non investigational medicinal product by MHRA meaning that giving the product to patients is not considered as a clinical trial. Just for transparency.

We have now added the following in the methods section:
This study was approved by the Bloomsbury ethics committee and all individuals provided informed written consent. Communication between the lead author (RWP) and the MHRA in 2019 confirmed that the study was not a Clinical Trial of an Investigational Medicinal Product (IMP) as defined by the EU Directive 2001/20/EC and no submission to the Clinical Trials Unit at the MHRA was required.

Reviewer #3 (Remarks to the Author):

This manuscript is greatly improved from the prior submission, and all of my original comments have been addressed. On the whole, this is a significant contribution to the field and I think worthy of publication. However, given the major revisions that have gone into this work, I have several new concerns that need to be addressed.

We thank the reviewer for their comments and positive assessment of our work.

It appears that the half-life of brain tau that was in the original publication (12 days) has been significantly adjusted (to 34 days). In the response to another reviewer's comments, the authors explain that this is due to an updated plasma leucine model that now incorporates more patient data. This is completely understandable and admirable that they are attempting to be as accurate as possible. However, this highlights how important the details of this model are to this major conclusion of the paper.

We are grateful that our attempts to diligently refine this model have been well received. The original submission in 2024 used a hypothetical leucine labelling protocol that was

greater than 1 g and proceeded for a longer time. This affected the early subjects who by definition received a lower dose of labelled leucine. This led to a sharp spike that then decayed exponentially through the two remaining data points. In the revision, we were able to come up with a much more logical cutoff for the hypothetical subject, targeting 1 g of labelled leucine to correspond to the normalization of all measurements by 1 g of labelled leucine. Additionally, the mean plateau value and mean values for the exponential decay were not available at the time of the first submission. We believe that the current model is optimal given the limited data because: the model has only one compartment and two parameters, and the plasma leucine curve is not only more accurate, it is more logically applied than before.

Perhaps I missed it, but I was unable to find a detailed description of the model and the assumptions that went into it in their methods. Given how important this is, this should be described in detail.

We have provided detail in the methods section regarding the model. The following text has been included:

The TTR data points were normalized by the dose of leucine in grams to account for differences in labelling period length. Tau 212-221 peptide was used for modelling as it provided the strongest and most consistent signal. Leucine labelling data was incomplete, so the leucine time course from over 100 human subjects was used as a surrogate. In this cohort, the mean plateau TTR of plasma leucine was 0.4199 during the 16-hour labelling period (4-16 h). This value was used as the plateau for this simulation. Because labelled leucine infusion was halted just prior to surgery for shunt placement, the total dose varied considerably. The measured tau TTR was thus normalized by the dose (i.e. TTR/g). To harmonize the model to this value, the length of labelling was set such that a hypothetical subject that was 80 kg would receive 1 g $^{13}C_6$-Leu, which corresponds to 0.25 days of labelling. Subject weights ranged from 70 – 112 kg with a median of 86.4 kg. Following cessation of the hypothetical 6 h infusion, the plasma leucine TTR was modelled as a triple exponential with the formula: Plasma Leucine TTR = 0.4199(0.3022*EXP(-38.32*(time – 0.25))+0.3022*EXP(-6.313*(time – 0.25))+0.0321*EXP(-0.0294*(time – 0.25))) with time in days. A one compartment model was used to fit the tau labelling data with a single scaling factor, with the leucine data as the input to the model, thus two parameters were fit with rate constant = 0.02064 and scaling factor 0.48. Modelling was performed in SAAM-II version 2.3.3 (Nanomath, Spokane, WA).*

In Figure 3B – the ventricular CSF group has a very wide spread, and only three datapoints; wouldn't it make sense to include more of your samples to make this more significant? I'm not sure how many of the 18 NPH patients in this study have ventricular CSF you can use, but if possible this would help buttress this finding.

We thank the reviewer for this suggestion. We have now included data from all available ventricular and lumbar CSF samples. There were 5 ventricular and 7 lumbar CSF samples that could be used. This improved the p value when comparing the compartmental difference using Mann-Whitney U test (p=0.030). Furthermore, we have now updated Supplementary Table 5 to account for the added specimens.

3) In Figure 4A – it seems odd to draw a curve based on two data points significantly beyond 0 (why didn't you do this for 4B?). To me these isolated points don't suggest a curve, but simply that the levels stay elevated for some time afterwords, with a suggestion of a later decline.

We recognize why the reviewer may have expressed concern about the decision to show a curve based on the data points available, particularly those at the later timepoints, but we seek to provide clarity.

For the early time points in Figure 4A, these provide guidance for the upslope of the labelling curve. A linear regression through the data reveals that these early time points do seem to contain useful and consistent information about the initial synthesis rate of tau. When the data is presented with a different scale (shown below, to reassure the editor), this becomes clearer, but we did not think this was essential to include in the manuscript.

While there are only two data points of significance for the later time points, these are rare and valuable data points that provide a unique insight into tau metabolism weeks after translation. The two individuals corresponding to these datapoints had a longer than usual time interval between SILK labelling and shunt insertion/brain biopsy due to clinical scheduling. It would not usually be ethically acceptable to delay surgery for research data collection and so these provided a rare opportunity to probe tau clearance/decay, and it would not be straightforward to increase the number of data points. We would say that the fit is in essence to eleven data points, nine that describe the initial upslope and the two later time points.

The full model fitting the early data points with the two later time points was done as minimally as possible, with a one compartment model and a single scaling factor. Scaling factors are consistently used in SILK studies to account for systematic mass spec errors that are present due to the challenge of measuring signal with extremely low amounts of labelled protein. In an ongoing unpublished study of 100 subjects having tau SILK (**ClinicalTrials.gov ID** NCT03938870) the mean scaling factor was 0.748 (full analysis ongoing).

We had less data available in 4b and so we did not fit a curve.

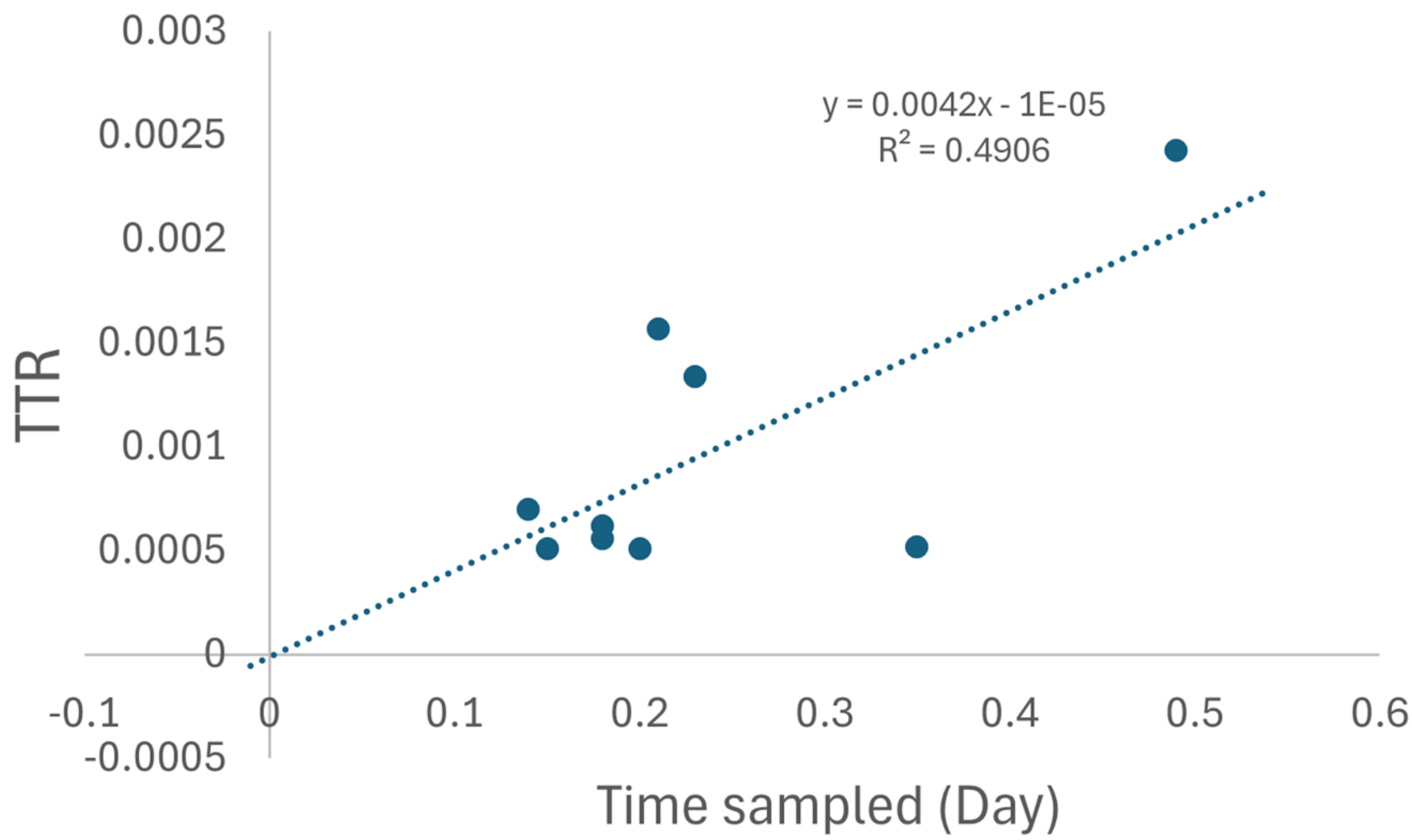

4) Lines 440-443 – A minor point, but I think contrasting the mechanism of beta-amyloid accumulation in NPH with all other non-NPH AD cases is an unnecessarily dichotomy. Beta-amyloid accumulation in AD has been proposed to be related to a range of processes including infection, brain trauma, ApoE4 physiology, and vascular dysfunction to give some examples (and then of course there is familial AD). I would simply say that altered CSF dynamics is likely one of many ways to contribute to beta-amyloid accumulation.

Thank you. We have now reworded that paragraph which now reads as follows:
It is possible that amyloid plaque accumulation in NPH may involve altered CSF dynamics as one of the several contributing mechanisms. Impaired bulk CSF clearance is well described in NPH (53, 98) and reduced Aβ clearance has also been implicated in AD (64). Given the overlap between these conditions (83), altered CSF dynamics may represent one pathway that contributes to Aβ accumulation and may help explain why patients with NPH developed co-existing proteinopathies.
