# Supplementary material for: Spatiotemporal characterization of Alzheimer disease pathology in living human brain tissue
Source: Nat Commun. 2026 Sep 24;17:9878. doi: 10.1038/s41467-026-76494-4 (PMC13612511; doi:10.1038/s41467-026-76494-4)
Supplement: Supplementary file 3 [file 41467_2026_76494_MOESM3_ESM.pdf]

nature portfolio

Corresponding author(s): Dr Aram Aslanyan and Dr Ross Paterson

Last updated by author(s): Jun 22, 2026

Reporting Summary

Nature Portfolio wishes to improve the reproducibility of the work that we publish. This form provides structure for consistency and transparency in reporting. For further information on Nature Portfolio policies, see our Editorial Policies and the Editorial Policy Checklist.

Statistics

For all statistical analyses, confirm that the following items are present in the figure legend, table legend, main text, or Methods section.

n/a	Confirmed	
☐	☒	The exact sample size (n) for each experimental group/condition, given as a discrete number and unit of measurement
☐	☒	A statement on whether measurements were taken from distinct samples or whether the same sample was measured repeatedly
☐	☒	The statistical test(s) used AND whether they are one- or two-sided<br>*Only common tests should be described solely by name; describe more complex techniques in the Methods section.*
☐	☒	A description of all covariates tested
☐	☒	A description of any assumptions or corrections, such as tests of normality and adjustment for multiple comparisons
☐	☒	A full description of the statistical parameters including central tendency (e.g. means) or other basic estimates (e.g. regression coefficient) AND variation (e.g. standard deviation) or associated estimates of uncertainty (e.g. confidence intervals)
☐	☒	For null hypothesis testing, the test statistic (e.g. F, t, r) with confidence intervals, effect sizes, degrees of freedom and P value noted<br>*Give P values as exact values whenever suitable.*
☒	☐	For Bayesian analysis, information on the choice of priors and Markov chain Monte Carlo settings
☒	☐	For hierarchical and complex designs, identification of the appropriate level for tests and full reporting of outcomes
☒	☐	Estimates of effect sizes (e.g. Cohen's d, Pearson's r), indicating how they were calculated

Our web collection on statistics for biologists contains articles on many of the points above.

Software and code

Policy information about availability of computer code

Data collection	Data was collected in Redcap (UCL central resource)
Data analysis	Described fully in methods section

For manuscripts utilizing custom algorithms or software that are central to the research but not yet described in published literature, software must be made available to editors and reviewers. We strongly encourage code deposition in a community repository (e.g. GitHub). See the Nature Portfolio guidelines for submitting code & software for further information.

Data

Policy information about availability of data

All manuscripts must include a data availability statement. This statement should provide the following information, where applicable:

- Accession codes, unique identifiers, or web links for publicly available datasets
- A description of any restrictions on data availability
- For clinical datasets or third party data, please ensure that the statement adheres to our policy

Data availability statement is provided in the manuscript.

Research involving human participants, their data, or biological material

Policy information about studies with human participants or human data. See also policy information about sex, gender (identity/presentation), and sexual orientation and race, ethnicity and racism.

Reporting on sex and gender	We have reported age range in order to protect participant identity given the small number of participants in this study. Sex is reported as biological sex.
Reporting on race, ethnicity, or other socially relevant groupings	N/A
Population characteristics	N/A
Recruitment	Participants were recruited sequentially as they presented to our clinical service.
Ethics oversight	This study was approved by the Bloomsbury Ethics Committee.

Note that full information on the approval of the study protocol must also be provided in the manuscript.

Field-specific reporting

Please select the one below that is the best fit for your research. If you are not sure, read the appropriate sections before making your selection.

- [x] Life sciences
- [ ] Behavioural & social sciences
- [ ] Ecological, evolutionary & environmental sciences

For a reference copy of the document with all sections, see nature.com/documents/nr-reporting-summary-flat.pdf

Life sciences study design

All studies must disclose on these points even when the disclosure is negative.

Sample size	This was a pilot study and so no formal power calculations were performed.
Data exclusions	Data exclusions have been described in the reporting summary.
Replication	Since the manuscript was originally submitted in 2024 we recruited an additional 12 individuals to replicate our findings. We replicated immunohistochemistry, isomerization of amyloid beta species, amyloid SILK, tau post translational modification quantitation and tau SILK and MALDI imaging and ventricular CSF SIMOA.
Randomization	The experiments were not randomized.
Blinding	Apart from the neuroradiologist who was blinded to biochemical and pathological findings, the rest of the investigators were not blinded to outcome assessments.

Reporting for specific materials, systems and methods

We require information from authors about some types of materials, experimental systems and methods used in many studies. Here, indicate whether each material, system or method listed is relevant to your study. If you are not sure if a list item applies to your research, read the appropriate section before selecting a response.

Materials & experimental systems

n/a	Involved in the study	
[ ]	[x]	Antibodies
[x]	[ ]	Eukaryotic cell lines
[x]	[ ]	Palaeontology and archaeology
[x]	[ ]	Animals and other organisms
[ ]	[x]	Clinical data
[x]	[ ]	Dual use research of concern
[x]	[ ]	Plants

Methods

n/a	Involved in the study	
[x]	[ ]	ChIP-seq
[x]	[ ]	Flow cytometry
[x]	[ ]	MRI-based neuroimaging

Antibodies

Antibodies used	6E10 and 4G8 (BioLegend), respectively binding to the N- and mid-terminal part of the Aβ peptide; using anti-beta-amyloid (10D5, Eli Lilly), anti-phosphorylated tau (PHF1, Feinstein Institute for Medical Research, Manhasset, NY), HJ8.5 and HJ8.7 antibody for tau; anti-phosphorylated alpha-synuclein (pSyn, Cell Applications), and anti-phosphorylated TAR DNA-binding protein of 43 kDa (pTDP-43, Cosmo Bio USA)
Validation	NA

Clinical data

Policy information about clinical studies

All manuscripts should comply with the ICMJE guidelines for publication of clinical research and a completed CONSORT checklist must be included with all submissions.

Clinical trial registration	NA
Study protocol	The study protocol is available
Data collection	Clinical data was entered into Redcap. Recruitment was between 2020 and 2025.
Outcomes	Observational

Plants

Seed stocks	NA
Novel plant genotypes	NA
Authentication	NA
